# Cell culture‐derived extracellular vesicles: Considerations for reporting cell culturing parameters

**DOI:** 10.1002/jex2.115

**Published:** 2023-10-16

**Authors:** Faezeh Shekari, Faisal J. Alibhai, Hossein Baharvand, Verena Börger, Stefania Bruno, Owen Davies, Bernd Giebel, Mario Gimona, Ghasem Hosseini Salekdeh, Lorena Martin‐Jaular, Suresh Mathivanan, Inge Nelissen, Esther Nolte‐’t Hoen, Lorraine O'Driscoll, Francesca Perut, Stefano Pluchino, Gabriella Pocsfalvi, Carlos Salomon, Carolina Soekmadji, Simon Staubach, Ana Claudia Torrecilhas, Ganesh Vilas Shelke, Tobias Tertel, Dandan Zhu, Clotilde Théry, Kenneth Witwer, Rienk Nieuwland

**Affiliations:** ^1^ Department of Stem Cells and Developmental Biology, Cell Science Research Center Royan Institute for Stem Cell Biology and Technology, ACECR Tehran Iran; ^2^ Advanced Therapy Medicinal Product Technology Development Center (ATMP‐TDC), Cell Science Research Center Royan Institute for Stem Cell Biology and Technology, ACECR Tehran Iran; ^3^ University Health Network Toronto Canada; ^4^ Department of Developmental Biology, School of Basic Sciences and Advanced Technologies in Biology University of Science and Culture Tehran Iran; ^5^ Institute for Transfusion Medicine University Hospital Essen, University of Duisburg‐Essen Essen Germany; ^6^ Department of Medical Sciences and Molecular Biotechnology Center University of Torino Turin Italy; ^7^ School of Sport, Exercise and Health Sciences Loughborough University Loughborough UK; ^8^ GMP Unit Spinal Cord Injury & Tissue Regeneration Centre Salzburg (SCI‐TReCS) and Research Program “Nanovesicular Therapies” Paracelsus Medical University Salzburg Austria; ^9^ Faculty of Natural Sciences Macquarie University North Ryde NSW Australia; ^10^ Institut Curie, INSERM U932 and Curie CoreTech Extracellular Vesicles PSL Research University Paris France; ^11^ Department of Biochemistry and Genetics, La Trobe Institute for Molecular Science La Trobe University Melbourne VIC Australia; ^12^ VITO (Flemish Institute for Technological Research), Health department Boeretang Belgium; ^13^ Department of Biomolecular Health Sciences, Faculty of Veterinary Medicine Utrecht University Utrecht The Netherlands; ^14^ School of Pharmacy and Pharmaceutical Sciences & Trinity Biomedical Sciences Institute Trinity College Dublin Dublin Ireland; ^15^ Biomedical Science and Technologies and Nanobiotechnology Lab IRCCS Istituto Ortopedico Rizzoli Bologna Italy; ^16^ Department of Clinical Neurosciences University of Cambridge Cambridge UK; ^17^ Institute of Biosciences and BioResources National Research Council Naples Italy; ^18^ Translational Extracellular Vesicles in Obstetrics and Gynae‐Oncology Group, UQ Centre for Clinical Research, Royal Brisbane and Women's Hospital, Faculty of Medicine The University of Queensland Brisbane Australia; ^19^ School of Biomedical Sciences, Faculty of Medicine University of Queensland Brisbane Australia; ^20^ Sartorius – BIA Separations Slovenia; ^21^ Laboratório de Imunologia Celular e Bioquímica de Fungos e Protozoários, Departamento de Ciências Farmacêuticas Universidade Federal de São Paulo (UNIFESP) SP Brazil; ^22^ Neurosciences and Cellular and Structural Biology Division, Eunice Kennedy Shriver National Institute of Child Health and Human Development National Institutes of Health Bethesda Maryland USA; ^23^ The Ritchie Centre Hudson Institute of Medical Research Clayton VIC Australia; ^24^ Departments of Molecular and Comparative Pathobiology and Neurology and Richman Family Precision Medicine Center of Excellence in Alzheimer's Disease Johns Hopkins University Baltimore Maryland USA; ^25^ Laboratory of Experimental Clinical Chemistry, Department of Clinical Chemistry, Amsterdam University Medical Centers Location AMC, University of Amsterdam Amsterdam The Netherlands; ^26^ Amsterdam Vesicle Center, Amsterdam University Medical Centers, location AMC University of Amsterdam Amsterdam The Netherlands

**Keywords:** cell culture‐conditioned medium, cellular therapy, ectosomes, exosomes, extracellular vesicles, in vitro, reproducibility, rigor, standardization

## Abstract

Cell culture‐conditioned medium (CCM) is a valuable source of extracellular vesicles (EVs) for basic scientific, therapeutic and diagnostic applications. Cell culturing parameters affect the biochemical composition, release and possibly the function of CCM‐derived EVs (CCM‐EV). The CCM‐EV task force of the Rigor and Standardization Subcommittee of the International Society for Extracellular Vesicles aims to identify relevant cell culturing parameters, describe their effects based on current knowledge, recommend reporting parameters and identify outstanding questions. While some recommendations are valid for all cell types, cell‐specific recommendations may need to be established for non‐mammalian sources, such as bacteria, yeast and plant cells. Current progress towards these goals is summarized in this perspective paper, along with a checklist to facilitate transparent reporting of cell culturing parameters to improve the reproducibility of CCM‐EV research.

## 1 INTRODUCTION

**FIGURE 1:**
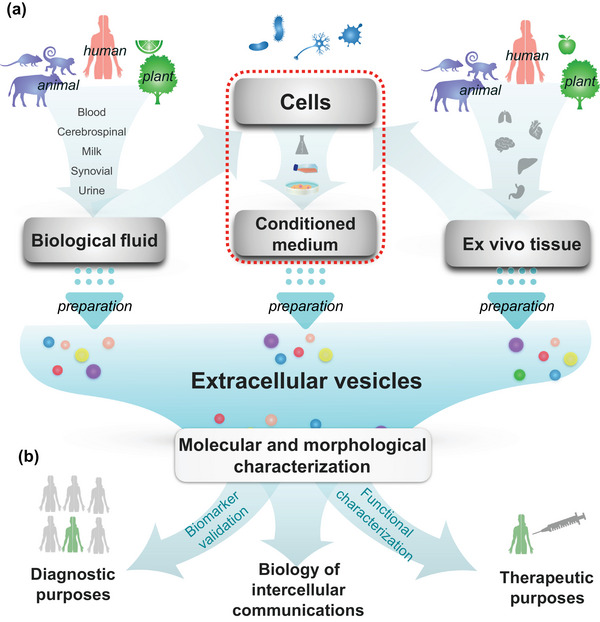
Source and application of extracellular vesicles. (a) Extracellular vesicles (EVs) can be prepared from biological fluids, cell culture‐conditioned medium (CCM), or ex vivo tissue. (b) Following molecular and morphological characterization, EVs can be used to study intercellular communication biology, to diagnose diseases (biomarker research), or as therapeutic agents (directly or as drug delivery vehicles). The dotted box indicates the focus of the CCM‐derived EV (CCM‐EV) task force on cell culturing parameters and their effects on EV release, content, and biological function.

Extracellular vesicles (EVs) are heterogenous membrane‐delimited particles that are released from cells in physiological and pathological states (Buzas, 2023; György et al., 2011; Witwer & Théry, 2019) and include endosome‐origin exosomes and plasma membrane‐shed ectosomes or microvesicles. EVs are composed of luminal cargo, a surrounding phospholipid membrane and membrane‐embedded and ‐attached macromolecular entities. A more loosely associated bio‐corona may also be of critical importance (Buzas, 2022; Wolf et al., 2022; Yerneni et al., 2022). In vivo, EVs are present in biological fluids such as blood, cerebrospinal fluid, saliva, tears and urine and in solid tissues. In vitro, EVs can be prepared from cell culture‐conditioned media (CCM; Figure 1a). EVs in CCM are released by the cultured cells but may also originate from supplements used for cell growth and/or differentiation. Since cells and medium supplements also release or contain non‐EV particles, it is often necessary to separate EVs from non‐EV components to identify EV‐specific contents and functions (Figure 1b).

Among the different sources of EVs, CCM is especially important because CCM‐derived EVs (CCM‐EV), whether native or engineered, can exert therapeutic functions. CCM‐EV have been administered to animals and patients to assess their therapeutic potential for several pathological conditions (Elahi et al., 2020; Kordelas et al., 2014; Kwon et al., 2020; Morse et al., 2005; Nassar et al., 2016; Park et al., 2021; Sengupta et al., 2020; Shekari et al., 2021; Warnecke et al., 2021). Table S1 gives an overview of clinical trials which have used or are currently using CCM‐EV from cellular sources such as dendritic cells, mesenchymal stromal cells (MSCs), tumor cells, T cells and plant cells. Producing cells, cell culture components, culture conditions and CCM collection and processing will impact the biochemical composition of EVs, association with other entities such as a co‐purifying bio‐corona, and biological function. However, there are currently no reporting guidelines for CCM‐derived EV.

To maximize the reliability and reproducibility of CCM‐EV research, transparent reporting is needed for the production and characterization of CCM‐EV. There are several guidelines on good practices for general cell and tissue culture for cellular therapy (Coecke et al., 2005; Pamies, 2018; Pamies et al., 2017). Furthermore, minimal criteria for reporting EV studies were proposed in MISEV2014 (Lötvall et al., 2014) and MISEV2018 (Théry et al., 2018), and a systematic assessment of articles published from 2012 to 2020 showed a clear association of study quality with citation of MISEV (Poupardin et al., 2021). In addition, working groups including members of the International Society for Extracellular Vesicles (ISEV), the International Society for Cell and Gene Therapy (ISCT), and the Society for Clinical, Research and Translation of Extracellular Vesicles Singapore (SOCRATES) have published best practice recommendations to produce MSC‐EVs and assays for therapeutic applications (Gimona et al., 2021; Pachler et al., 2017; Rohde et al., 2019; Witwer et al., 2019).

The ISEV Rigor and Standardization (R&S) Subcommittee (Nieuwland et al., 2020) aims to improve reproducibility of EV research (Börger et al., 2020; Clayton et al., 2019; Erdbrügger et al., 2021; Welsh et al., 2020). Although MISEV2018 provides mandatory reporting regarding CCM (Théry et al., 2018), and the importance of culturing parameters for the production of EVs is commonly recognized (Hill et al., 2013; Gudbergsson et al., 2016; Gurunathan et al., 2021; Patel et al., 2017, 2018; Staubach et al., 2021), standardized reporting of culturing parameters has not been implemented in most publications. Therefore, the R&S subcommittee established a task force on CCM‐EV to focus on cell culturing parameters and their effects on EVs (Figure 1a, red dotted box). Here, the CCM‐EV task force focuses on selected cell monoculture parameters that may affect EV characteristics, gives recommendations for transparent reporting, and identifies open questions. Our goals are to increase awareness and promote reproducibility in EV research when CCM is the starting material.

## 2 CELL CULTURE PARAMETERS

### 2.1 Cells (source cells)

#### 2.1.1 Cell identity

The identity of the donor/parental cell is a major determinant of EV yield, phenotype and biological function. Different cells may have different basal rates of EV production. For example, EV yield and function may differ between MSCs derived from different donors and tissue sources (Almeria et al., 2022; Fafián‐Labora et al., 2017; Kang et al., 2016; Komaki et al., 2017; Mendt et al., 2018; Merckx et al., 2020; Nakamura et al., 2015; Rosenberger et al., 2019; Teng et al., 2015; Tracy et al., 2019). Also, different cancer cell lines produce different numbers of EVs (Charoenviriyakul et al., 2017; Hurwitz et al., 2016; Salomon et al., 2014). For cell lines, regular short tandem repeat (STR) profiling can confirm identity of cells and detect cellular cross‐contamination (American Type Culture Collection Standards Development Organization Workgroup ASN‐000, 2010; Barallon et al., 2010; Masters et al., 2001; Reid et al., 2004).

##### 2.1.1.1 Recommendations

- Report the identity of EV‐producing cells according to consensus in the research communities that regularly use such cells. For example, ISCT criteria define multipotent MSCs by positive and negative marker expression and differentiation potential (Dominici et al., 2006; Viswanathan et al., 2019; Witwer et al., 2019). Criteria for clinical‐grade human induced pluripotent stem cell lines have also been established (Sullivan et al., 2018). Additional information regarding specific cell lines can be found at https://www.atcc.org/, and cell‐line specific molecular information is available at https://depmap.org/portal.
- If downstream EV analysis focuses on a particular marker, confirm that the EV marker of interest is also present in/on the cultured cells and thus likely originates from those cells and not from another source.

##### 2.1.1.2 Outstanding questions

- In some cell monocultures, multiple populations of cells are nevertheless present (Costa et al., 2021; Sato et al., 2016; Wang et al., 2021) (i.e., cells with genetic, epigenetic, morphologic or functional differences), which contributes to the heterogeneity of EVs and makes it more difficult to interpret from which cell types the detected EVs are derived. How can we reduce or control the heterogeneity of source cells?

#### 2.1.2 Characteristics of the cell/tissue donor

The potency of primary cells may be affected by characteristics of the cell donor, including overall health status, age (Choudhery et al., 2014; Dorronsoro et al., 2021; Gruber et al., 2012; Katsara et al., 2011; Payr et al., 2020; Sarkar et al., 2018; Siegel et al., 2013), biological sex (Katsara et al., 2011; Sammour et al., 2016; Siegel et al., 2013), pregnancy (Li et al., 2017; Mathew & Bhonde, 2017; Xia et al., 2007; Zhu et al., 2020), medication intake, nutritional status, infection and microbiota. In turn, these factors may influence the release, composition and function of EVs. For example, functional comparison of EVs from young and old donors revealed donor age dependencies (Agarwal et al., 2017; Sun et al., 2020; Umezu et al., 2017). The biochemical composition of EVs derived from different human donors also varies, possibly due to donor characteristics (Kim et al., 2016; Kordelas et al., 2014).

##### 2.1.2.1 Recommendations

- Report (if available) known donor characteristics, including but not limited to biological sex, age, chronic diseases, infection status, medication and pregnancy/complications.
- Screen cells for the presence of infectious agents, especially if clinical applications are expected. Note that some viruses can integrate into the genome.
- Report pre‐culture processing of the cells.

#### 2.1.3 Initial seeding density

The initial concentration, or the number of cells per volume or surface area in the culture flask or plate, affects cell growth and differentiation, as well as the release of EVs. This dependence was shown for MSCs and cancer cells (Ludwig et al., 2019; Patel et al., 2017).

##### 2.1.3.1 Recommendations

- Report the initial seeding density of cells, ideally as cells per cm^2^ (for adherent cells) or cells per mL (for suspension cells).
- Reduce variation by using the same seeding concentration and cell splitting intervals across experiments.
- Optimal plating density has been reported for specific cells and culturing conditions (Sotiropoulou et al., 2006); otherwise, optimize seeding density.

#### 2.1.4 Cell density or confluency of cells at the time of CCM collection

The density of cells affects the cell physiology (Balint et al., 2015; Bereiter‐Hahn et al., 1998; Jacobs et al., 2016; Ren et al., 2015; Sekiya et al., 2002; Trajkovic et al., 2019), thereby affecting the production rate, biochemical composition and function of released EVs (Cao et al., 2020; Gao et al., 2020; Guerreiro et al., 2018; Gurunathan et al., 2021; Haraszti et al., 2018; Hayes et al., 2005; Mitchell et al., 2008; Palviainen et al., 2019; Rocha et al., 2019; Sekiya et al., 2002; Steinman et al., 2003; Thippabhotla et al., 2019; Yan & Wu, 2020; Yang et al., 2020). In over‐confluent cell cultures, the therapeutic potential of MSCs may change due to contact‐inhibited growth or contact‐induced senescence (Balint et al., 2015; Ho et al., 2011; Sekiya et al., 2002). For 2D cultures, the percentage of surface area covered can be reported, but this metric may be inadequate, as cells are not equally large.

##### 2.1.4.1 Recommendations

- Count cell numbers in a standardized procedure (e.g., with technical replicates) before seeding and after final harvesting at a given passage.
- Report cell density at the time of CCM collection (cells/cm^2^ or cells/mL). For 3D cultures of spheroids or organoids, the reporting parameter might be the diameter of the spheroids or another appropriate measure.

##### 2.1.4.2 Outstanding questions

- In 2D cultures, the term ‘confluency’ is often used. However, this remains a largely ‘qualitative’ parameter, and measurements may differ between individual researchers and laboratories due to different equipment and procedures. More objective measurements may be achieved by preparing and following detailed standard operating procedures that help to improve reproducibility.

#### 2.1.5 Cell sub‐culturing, population doubling and passage number

Transferring cultured cells from high density to low density for propagation (‘sub‐culturing’, ‘passaging’, ‘splitting’ or ‘re‐plating’) can influence cellular properties including the expression of cell surface markers, senescence and genetic stability (Kassem et al., 1997; Meza‐Zepeda et al., 2008; Wagner et al., 2008; Yang et al., 2018). Suspension cells are either simply diluted into fresh culture medium, or, in some cases, the whole medium is replaced by fresh medium, following a low‐speed centrifugation step. However, adherent cells regularly must be detached from vessel of carrier surfaces with the help of enzymes and/or mechanical intervention before passaging, and detachment methods may affect the cells and their membrane. For example, human embryonic stem cells experienced genetic instability based on the sub‐culturing method (Bai et al., 2015; Garitaonandia et al., 2015). The passage time is directly related to the population doubling time of a given cell culture. If primary cells are used, senescence is dictated by the total achievable population doubling number (Hayflick limit) (Shay & Wright, 2000) and depends on cell type, donors and culture conditions (Meza‐Zepeda et al., 2008). EVs from different passage numbers of the same cells may differ in size, concentration and functions (Beer et al., 2015; Boulestreau et al., 2020; Dorronsoro et al., 2021; Fafián‐Labora et al., 2017; Lehmann et al., 2008; Lei et al., 2017; Patel et al., 2017, 2018; Sarkar et al., 2018; Takahashi et al., 2017; Takasugi et al., 2017; Venugopal et al., 2017).

##### 2.1.5.1 Recommendations

- Report passage number, starting from the original stock and consider Master and Working Cell Bank passage numbers. It is recommended to consider the maximum number of passages that primary cells or cancer cells can undergo without changing their genotype and/or phenotype.
- For adherent cells, indicate the method of passaging with details necessary for exact replication, including but not limited to the type of treatment (e.g., trypsin or EDTA with any commercial name, mechanical, or any other methods), the concentration of any reagents, time of treatment (in minutes), temperature of incubation and if/how any enzymatic treatments are stopped.
- Report recovery time, if any, between sub‐culturing and the start of EV collection.
- Consider avoiding the application of undefined animal‐derived products during sub‐culturing processes, the use of recombinant enzymes is preferable.

##### 2.1.5.2 Outstanding questions

- To what extent does the effect of passage number and senescence differ between culture conditions, including static versus bioreactor, and adherent versus free‐floating?

#### 2.1.6 Cell viability

In contrast to healthy cells, dying cells may preferentially release EV subtypes such as apoptotic bodies and necroptotic bodies, although these may also have therapeutic activity (Atkin‐Smith et al., 2015; Battistelli & Falcieri, 2020; Baxter et al., 2019; Brock et al., 2019; Caruso & Poon, 2018; Crescitelli et al., 2013; Dieudé et al., 2015; Galluzzi et al., 2018; Gregory & Dransfield, 2018; Kakarla et al., 2020; Lázaro‐Ibáñez et al., 2014; Li et al., 2020; Liu et al., 2020; Park et al., 2018; Phan et al., 2020; Poon et al., 2019; Shlomovitz et al., 2021; Théry et al., 2001; Zheng et al., 2021). Since the proportion of live and dying/dead cells affects the proportion of EV subtypes in CCM, it is important to assess the viability of the cells as well as the contribution of dying or dead cells at the time of CCM collection. Cell death is commonly estimated using membrane permeability‐based stains or metabolic assays. In bioreactors, metabolic readouts such as glucose and lactate provide insight throughout the cell culture (Mendt et al., 2018). However, only living cells contribute to metabolism, and these methods do not indicate the percentage of apoptotic or dying cells. An early ISEV position paper recommended that cell death percentage in culture should be less than 5% (Witwer et al., 2013); however, achieving 95% viability is not always possible—for example, in drug response studies—and assessing viability is also not always immediately possible, such as with some types of bioreactors and in multiple‐ or continuous‐harvest 2D systems.

##### 2.1.6.1 Recommendations

- Wherever possible, report the percentage of viable cells at the time of CCM collection.
- Wherever possible, document cellular morphology by taking representative images when harvesting.

##### 2.1.6.2 Outstanding questions

- Can we understand the contribution of dying cell‐derived EVs to the overall biological and potential therapeutic activity in a given EV preparation?
- Can we develop monitoring systems, for example, glucose consumption or lactate production, for 2D and 3D cell expansion systems?

## 3 EV PRODUCTION MEDIUM

Basal cell culture medium contains nutrients, especially glucose (as the main source of energy), and amino acids, and can be supplemented with serum or other components. The EV production medium (the medium used for EV isolation) may be different from the basal cell culture medium.

### 3.1 Basal medium, glucose and amino acids

Commonly used basal media for mammalian cell cultures, such as Roswell Park Memorial Institute (RPMI), Dulbecco's Modified Eagle Medium (DMEM), and alpha‐modified Minimal Essential Medium (MEM), contain inorganic salts (sodium, ferric, magnesium and potassium salts), and micronutrients (vitamins and minerals). Any of these components may affect cell growth and differentiation, and EVs (Arigony et al., 2013; Arodin Selenius et al., 2019; Bhat et al., 2021; Kawakami et al., 2016; Watchrarat et al., 2017; Wu et al., 2009; Zhu et al., 2021).

D‐glucose is the major carbon source in the cell culture growth medium. Increased release of EVs has been observed in the presence of both elevated and reduced glucose levels, and this effect seems cell type‐dependent (Burger et al., 2017; Garcia et al., 2015; Rice et al., 2015; Thom et al., 2017). Several reports have shown a change in the biochemical composition or biological function of EVs when producing cells cultured in a medium containing high concentrations of glucose (Davidson et al., 2018; De Jong et al., 2012; Huang et al., 2020; Lin et al., 2019; Thom et al., 2017; Wu et al., 2017; Zhu et al., 2019; Zhou et al., 2021).

Amino acids and proteins are required for cell growth. The effects of glutamine and leucine on cell proliferation and EV biogenesis have been documented (Dai et al., 2015; Fan et al., 2020; Kim et al., 2017; Rubin, 2019; Zhao et al., 2021). If stabilized versions of amino acids are used as supplements, a different concentration of this amino acid in the medium over time will be maintained than when using the native version. Moreover, free amino acids present in the CM may become incorporated into EVs.

#### 3.1.1 Recommendations

- Report the basal medium used, including the catalog number.
- Report the nature and concentration of additives added during cell culture.

### 3.2 Antibiotics and antimycotics

Antibiotics affect cell characteristics (marker expression) and function, and may also change the biochemical composition and function of EVs (Cohen et al., 2006; Llobet et al., 2015; Ryu et al., 2017; Skubis et al., 2017). Similarly, antimycotics may also change the production and composition of EVs (Németh et al., 2017).

#### 3.2.1 Recommendations

- Report the use of antibiotics and antimycotics, including if they are used during pre‐conditioning and/or conditioning steps.

#### 3.2.2 Outstanding questions

- What are the effects of antibiotics and antimycotics on the biochemical composition and function of EVs?
- If packaged into or associated with EVs, how do antibiotics and antimycotics affect the therapeutic function of EVs?

### 3.3 Complex biological supplements

The most commonly used complex biological supplements are serum and platelet lysate (PL). Serum, often fetal bovine serum (FBS), also known as fetal calf serum (FCS), provides essential components such as growth factors for cell proliferation and differentiation. However, FBS also contains (bovine‐derived) EVs and non‐EV particles such as lipoproteins (Brunner, 2010; Fisher et al., 1958; Gstraunthaler, 2003; Palviainen et al., 2020; Subbiahanadar Chelladurai et al., 2021).

Platelet lysate (PL) has effects comparable or superior to serum in some cell cultures (Hemeda et al., 2014), and, if harvested from donors of the same species as the cultured cells, PL may be used as a xenogeneic‐free serum substitute (Becherucci et al., 2018; Bianchetti et al., 2021; Capelli et al., 2007; De Almeida Fuzeta et al., 2020; Gottipamula et al., 2012; Griffiths et al., 2013; Hemeda et al., 2014; Johansson et al., 2003; Juhl et al., 2016; Menard et al., 2013; Oeller et al., 2021). Like serum, also PL is rich in growth factors and may contain pathogens like viruses, and may exhibit donor‐to‐donor variability (Burnouf, 2018; Guiotto et al., 2020; Hawkes, 2015; Henschler et al., 2019; Hemeda et al., 2014; Schallmoser et al., 2020). Pathogen inactivation or depletion by irradiation, with or without the addition of photoactive chemicals or filtration (Oeller et al., 2021; Organization, 2004; Schallmoser et al., 2020; Viau et al., 2019) is required for regulatory compliance. PL also contains components secreted from platelets such as EVs, coagulation proteins and growth factors, which may contribute to its proliferation‐supporting activity (Torreggiani et al., 2014) (reviewed in Antich‐Rosselló et al., 2021; Kerris et al., 2020; Melki et al., 2017).

A position statement from the working group on cellular therapies of the International Society of Blood Transfusion (ISBT) discussed human PL (hPL) production, manufacturing, and quality management (Schallmoser et al., 2020), and the barriers to the translational use of hPL have been discussed in a joint publication of the Association for the Advancement of Blood and Biotherapies (AABB) and ISCT (Bieback et al., 2019). The use of hPL reduces the problem of animal components, although the use of PL for the manufacturing of therapeutic EVs requires specific precautions and pre‐processing steps.

The presence of coagulation factors and fibrinogen in PL can lead to fibrin precipitates during the cell expansion process, which may be incompatible with processes such as filtration. Addition of heparin to the growth medium inhibits fibrin formation. Alternatively, addition of calcium chloride to PL may trigger coagulation and fibrin formation (Staubach et al., 2021), and the fibrin clot that is formed can be removed by centrifugation prior to use.

#### 3.3.1 Recommendations

- Report the percentage, producing company (city, country), and catalog number of complex biological additives including sera.
- Report the source and percentage of PL used at the various steps of cell expansion and CCM production.
- Report the process of PL production, including fibrin depletion methods and pathogen inactivation.
- Report the concentration of heparin present in the growth medium, and consider the potential adverse effects of heparin on separation and downstream analysis of EVs (Atai et al., 2013; Beutler et al., 1990).

### 3.4 EV depletion

Serum and PL contain EVs, DNA fragments, non‐EV particles such as protein aggregates and lipoproteins, and micronutrients (Arigony et al., 2013; Lehrich et al., 2021; Urzì et al., 2022), which may complicate the isolation, assessment, and interpretation of the biochemical composition and function of CCM EVs. For example, FBS‐derived EVs or particles may co‐isolate with EVs produced by cultured cells (Lehrich et al., 2021). Various methods have been described to deplete EVs from the serum (Driedonks et al., 2019; Kim et al., 2021; Kornilov et al., 2018; Lehrich et al., 2018; Liao et al., 2019, 2017; Mannerstrom et al., 2019). However, these methods do not remove all or exclusively EVs (Kornilov et al., 2018; Lehrich et al., 2018; Shelke et al., 2014; Tosar et al., 2017), and most studies do not report the degree of depletion. The biochemical composition and function of EVs released by cells cultured in the presence of EV‐depleted serum may not be identical to those released in the presence of EV‐containing serum. This is possibly due to the fact that serum‐derived EVs themselves have multiple effects on cultured cells (Beninson & Fleshner, 2015; Cavallari et al., 2017; Gu et al., 2018; Ochieng et al., 2009; Urzì et al., 2022).

#### 3.4.1 Recommendations

- When serum is diluted prior to removal of EVs, indicate whether, how (fold dilution), and with what (buffer, medium) the serum was diluted.
- For ultracentrifugation‐based depletion of EVs, report the centrifugation speed and time, rotor specifications (K value, angle, tube volume), and temperature.
- For tangential flow filtration‐based depletion of EVs, report the details including membrane/device manufacturer, material type, pore size, filtration surface area, flow rate, and temperature.
- Monitor and report the level of EV depletion by comparing pre‐and post‐depletion material

### 3.5 Serum‐free culturing

Considering the complex nature and important functions of serum in cell culture media, any manipulation including replacing the medium with a serum‐free medium, or heat treatment to inactivate the complement system, may alter the physiology of the cultured cells and derivative EVs (Alcolea et al., 2016; Angelini et al., 2016; Aswad et al., 2016; Bhat et al., 2021; Cavallari et al., 2017; Clabaut et al., 2015; Eitan et al., 2015; Frigerio et al., 2021; Lehrich et al., 2021, 2018; Li et al., 2015; Liao et al., 2017; Mehta et al., 2010; Obrochta et al., 2015; Pham et al., 2021; Urzì et al., 2023). Some reports showed that serum‐free or chemically defined medium enhance EV production (Biadglegne et al., 2021; Bost et al., 2022; Faruqu et al., 2021; Figuero, 2021).

#### 3.5.1 Recommendations

- In the case of serum‐free media, report if no replacement supplement was added (e.g., in ‘starvation’ experiments), or if replacement supplements were added that are nutritionally complete.
- Report the time duration of cell culture without serum or other complex additives.
- Document any observed change in cellular morphology or characteristics.
- Quantify the number of particles in non‐conditioned medium using the same process as for conditioned medium at least once.

#### 3.5.2 Outstanding questions

- EV depletion from serum may result in the removal of non‐EV components such as lipids and proteins. Does EV‐depleted growth medium support cell proliferation to the same extent as a non‐EV depleted serum‐containing medium?
- The efficiency of EV depletion methods varies. Monitoring the efficiency of EV depletion is difficult because none of the currently available EV detection methods can detect all EVs. Performing a procedural control, that is, comparing the negative control of the same volume of complete, unconditioned medium processed the same way as CCM, is a functional alternative, but will not fully answer the question of depletion efficiency. How can we best report the efficiency of EV depletion?

### 3.6 Cell (pre)conditioning

Other factors used to promote proliferation, differentiation, silencing, or priming of cells, and/or to otherwise manipulate cells and their functions, may influence the release of EVs (Bai et al., 2020; Emam et al., 2018; Martens‐Uzunova et al., 2021; Pecan et al., 2020; Savina et al., 2003; Segura et al., 2005; Soekmadji et al., 2017; Taylor et al., 2017; Wang et al., 2020) or their therapeutic potential (Akbar et al., 2017; Cossetti et al., 2014; De Jong et al., 2012; Hosseinkhani et al., 2017, 2020; Lopatina et al., 2014; Patel et al., 2019; Peltzer et al., 2020; Sutter et al., 2021; Varkouhi et al., 2019; Yang et al., 2020; Zhang et al., 2018, 2020).

#### 3.6.1 Recommendations

- Report the concentration, company (city, country), and catalog number of small molecules, cytokines/chemokines, or other RNA/protein/lipid‐based cell‐modulatory reagents. Also indicate if they are used during pre‐conditioning and/or medium‐conditioning periods.

#### 3.6.2 Outstanding questions

- Do cell‐modifying factors associate with EVs or non‐EV particles, and if so, what are their possible effects?

## 4 CULTURE CONDITIONS

Changes in biophysical and biochemical cell culture conditions may affect cell growth and the release and biochemical composition of EVs. External culturing conditions include the concentration of gasses, 2D or 3D culturing, pH, temperature, physical stimulus, the composition of culture vessels/substrates, and in‐experiment manipulations of the cell culture medium.

### 4.1 Gas conditioning

The effect of oxygen concentration on the biochemical composition, function, and release of EVs is well studied (Agarwal et al., 2017; Alharbi et al., 2021; Almeria et al., 2019; Davidson et al., 2018; De Jong et al., 2012; Dougherty et al., 2020; Dutta et al., 2020; King et al., 2012; Kucharzewska et al., 2013; Kumar & Deep, 2020; Salomon, Kobayashi et al., 2013; Salomon, Ryan et al., 2013; Xue et al., 2018; Zhang et al., 2017). So‐called ‘normoxia’, that is, 21% O_2_ or ‘atmospheric’ oxygen, is in fact hyperoxia for most mammalian cell types. Even in lung alveoli, the oxygen level is 14.5%, and at the venous end of circulation, oxygenation is around 6.5% (Mckeown, 2014). Depending on the exact tissue type and anatomy, physiological hypoxia may be in the range of 2%−6% oxygen, and pathological hypoxia may be even lower, ranging from 0.3% to 4.2% oxygen (Mckeown, 2014). Cells cultured under atmospheric conditions have thus been exposed or even adapted to higher‐than‐physiologic oxygen concentrations. To reach true normoxia, specialized culture chambers are required.

#### 4.1.1 Recommendation

- Report gas concentrations used during cell culture, especially of oxygen, but where applicable also of CO_2_, and nitrogen, as well as any interruptions in the gas conditions during the culture process.

### 4.2 2D or 3D cultures: vessels, coatings, matrices

Some reports have shown that cells cultured in 3D systems typically produce more EVs compared with conventional 2D cultures or adherent cultures (Cao et al., 2020; De Almeida Fuzeta et al., 2020; Grangier et al., 2021; Guo et al., 2021; Haraszti et al., 2018; Kim et al., 2021; Patel et al., 2018; Phan et al., 2019, 2018; Rocha et al., 2019; Watson et al., 2016; Yan & Wu, 2019; Yan et al., 2018; Yang et al., 2020; Zhang et al., 2021). On the other hand, 3D system of cultures can lead to different biochemical compositions and functions of EVs compared with those obtained with 2D systems (Cao et al., 2020; Gao et al., 2020; Haraszti et al., 2018; Jarmalavičiūtė et al., 2015; Kim et al., 2021; Miceli et al., 2019; Rocha et al., 2019; Thippabhotla et al., 2019; Yan & Wu, 2019, 2020; Yang et al., 2019). The materials from which cell culture vessels (e.g., flasks or plates) are, as well as any support matrices, such as bead substrates and vessel coatings, may affect EV characteristics and should be reported accordingly (Jangamreddy et al., 2018; Park et al., 2020; Sutter et al., 2021; Szvicsek et al., 2019; Wu et al., 2021).

#### 4.2.1 Recommendations

- Report all culturing conditions, including, but not limited to, surface area, volume, and preconditioning with buffers.
- Report the composition of any support matrix, including, for example, substrate beads and vessel coatings.
- Indicate the procedure used to prepare and apply any support matrix.

### 4.3 pH

The pH of the medium affects release, uptake and biochemical composition of EVs (Ban et al., 2015; Boussadia et al., 2018; Cheng et al., 2019; Logozzi et al., 2018; Parolini et al., 2009; Perut et al., 2019; Nakase et al., 2021). Medium pH affects the stability of released EVs (Cheng et al., 2019). Although we are not aware of reports on the effects of pH on generation of therapeutic EVs, changes in pH influence stem cell reprogramming or differentiation (Fliefel et al., 2016; Kim, 2021; Massa et al., 2017).

#### 4.3.1 Recommendations

- Monitor and report the pH of medium.
- Report if cells were grown using a pH buffering agent, and, if so, its concentration.

#### 4.3.2 Outstanding questions

- How does pH affect the production and composition of EVs?

### 4.4 Physical stimulation

Applying physical stimuli such as electrical stimulation, acoustic irradiation, and mechanical forces (including shear and stretching of the scaffold) may increase the production and/or change the biochemical composition of EVs (Ambattu et al., 2020; Eichholz et al., 2020; Fukuta et al., 2020; Guo et al., 2021; Hergenreider et al., 2012; Letsiou et al., 2015; Maeshige et al., 2021; Morrell et al., 2018; Ridger et al., 2017; Vion et al., 2013; Xia et al., 2020; Yang et al., 2017; Zeng et al., 2019; Zhao et al., 2020). Moreover, for some cell types, temperature may affect EV release and uptake dynamics and may also change EV function (Mahmood et al., 2023; Otsuka et al., 2020).

#### 4.4.1 Recommendation

- Report all applied physical stimuli in detail, including, but not limited to, intensity, voltage, time and temperature.

### 4.5 Replacement of medium

Medium replacement during cell culturing keeps the cells healthy by providing fresh nutrients and eliminating waste products. However, during medium replacement, the secretome of cells including EVs is removed, and cells start to secrete new EVs (Patel et al., 2017; Vis et al., 2020).

#### 4.5.1 Recommendations

- Report the medium replacement protocol, including what percentage of the volume is replaced, at what interval (in hours) the medium is changed or replenished, intermediate washing steps (if any), changes to the medium composition, and continuous‐flow feeding (with recirculation or not).
- In some cases, different media formulations are used at various stages of an experiment. If so, indicate when and how, as well as the details of each formulation and why each specific formulation was used.

#### 4.5.2 Outstanding questions

- What are the effects of maintaining cells in the same medium for extended durations and of medium replacement on EV release, biochemical composition, and function?

## 5 COLLECTION (HARVEST) OF CCM

Duration and frequency of CCM collection may affect the yield and surface protein expression of EVs (Mendt et al., 2018; Patel et al., 2017). There are three main approaches to CCM harvest: single harvest, multiple harvests, and continuous harvest. The duration of conditioning differs between studies and assays from hours to days. During conditioning, cells continuously release and uptake EVs; therefore, the time interval during which cells are cultured affects the biochemical composition, function, and release of EVs due to changes in the release and possible re‐uptake of EVs, along with cell growth and differentiation (; Davidson et al., 2018; Flores‐Bellver et al., 2021; Kim et al., 2016; Li et al., 2015).

### 5.1 Recommendations

- For a single harvest: report the total time (duration) of conditioning.
- For multiple harvests: report the number of harvests, duration of conditioning for each, and whether all or some fraction of medium is collected (i.e., complete or partial medium replacement), specifying the fraction where applicable.
- Report how many (if any) of the multiple harvests are pooled.
- Report on any pre‐pooling analyses and inclusion criteria.
- For continuous harvest, report the duration of continuous collection and harvest rate (i.e., volume per unit time).
- Report the duration of conditioning and keep it constant across experimental repeats.

### 5.2 Outstanding questions

- How do continuous harvest and multiple harvests affect EV release, biochemical composition and function?
- Since cells release EVs into and simultaneously uptake EVs from CCM, cultures may reach an equilibrium between EV release and uptake, and this may occur at different times for different cultures and conditions. What are the effects of conditioning time on EV release, biochemical composition and function?

## 6 MICROBIAL CONTAMINANTS OF CCM

CCM may be contaminated with viruses, bacteria, fungi, and other unwanted bioactive components such as endotoxins. Active bacterial and fungal contaminations are often apparent in culture and in stored EV samples by visual examination or aided by light microscopy, in which case such materials can be discarded. However, many contaminations are not easily detected. The presence of Mycoplasma, for example, may not be readily apparent, and Mycoplasma species can release EVs into CCM (Gaurivaud et al., 2018). Mycoplasma‐infected cells release EVs that differ in function from EVs released by non‐infected cells (Cronemberger‐Andrade et al., 2020; Quah & O'neill, 2007; Yang et al., 2012). Bacterial or fungal‐derived EVs and other bioactive components such as endotoxins may also be introduced into CCM from raw materials or culture vessels even if actively replicating organisms are not present. Viruses and viral components may also be present in cells and/or culture components and may cause unintended effects (Barone et al., 2020; Merten, 2002). Contaminants such as Mycoplasma and viruses may not be eliminated by 0.22‐micron filtration of culture medium, conventionally used to eliminate active bacterial contamination.

### 6.1 Recommendations

- Report if any screening of Mycoplasma or other contaminants in parental cell lines/cultures was done and the results thereof. Report details of any antimicrobial treatment.
- Especially if EVs are administered to animals or humans, measure and report the level of endotoxins in prepared EVs according to international pharmacopoeia (USP<85>, EP 2.6.14, JP 4.01) (Commission, E.P., E.D.f.t.Q.o 2010; Revision, U.S.P.C.C.o 2008; The Japanese pharmacopoeia 1996), and provide evidence of sterility of prepared EVs according to international pharmacopoeia (USP<71>, EP 2.6.1, JP 4.06) (Commission, E.P., E.D.f.t.Q.o 2010; Revision, U.S.P.C.C.o 2008; The Japanese pharmacopoeia 1996).

## 7 DISCUSSION

**FIGURE 2:**
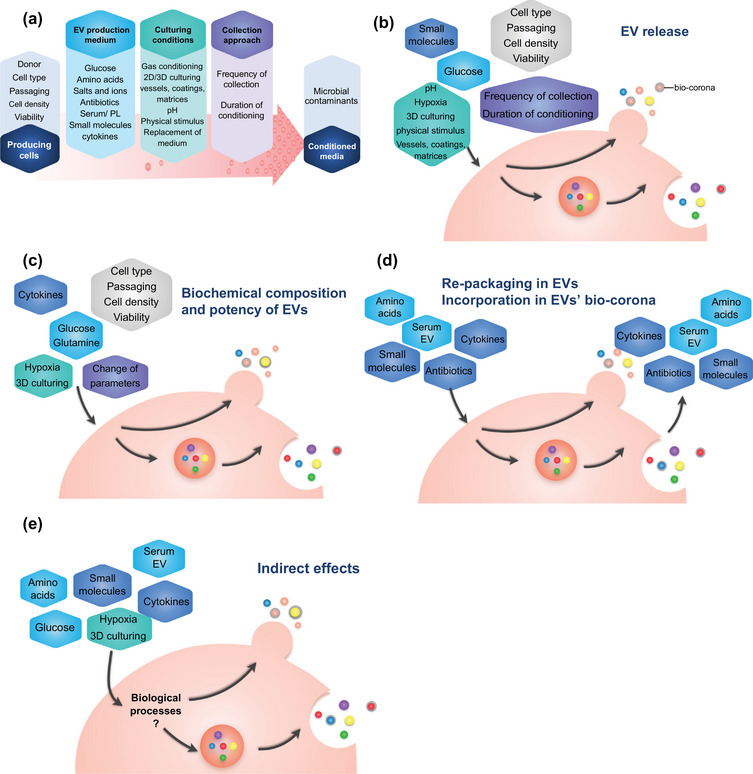
Cell culture parameters and their effects on production of extracellular vesicles. (a) All parameters affect cells during conditioning and release of extracellular vesicles (EVs). (b) Parameters influencing release of EVs, and (c) biochemical composition of EVs. (d) Cells might also re‐package cell culture medium supplement components into/onto released EVs. (e) Cell culturing parameters may affect processes that indirectly affect EVs.

In this perspective paper, we aim to raise awareness of the effects of cell culturing parameters (Figure 2a) that may affect EV release (Figure 2b), biochemical composition, and/or function (Figure 2c). Cells can ingest components that are present in the media and re‐package them into or onto EVs (Figure 2d). Moreover, cell culturing parameters can influence cellular processes other than EV biogenesis that indirectly affect EVs (Figure 2e). We have compiled a checklist of these parameters in Table 1. The aim of the checklist is to collect as much information as possible to improve reproducibility. The information can be summarized in the Methods section, and the checklist can be added as supplementary information to the manuscript. We are aware that some recommendations may be harder to implement than others. For instance, measuring pH of a culture medium at different stages of the culture is not a routine process; thus, it may not become readily or routinely implemented. At present, multiple task forces and working groups have developed or are developing checklists, and the goal of ISEV is to develop on online repository, either as a stand‐alone or combined and integrated with an already existing platform such as EV‐TRACK. Such an online repository will be useful to re‐evaluate collected data and can be used to update future recommendations accordingly.

**TABLE 1:** Cell culturing parameter checklist.

Cell culturing parameters		Items	
**1. Producing cells (source cells)**	**1.1 Cell identity**	Name of cell Origin (e.g. donor or cell bank) Cell type identification	
	**1.2 Cell donor characteristics**	Biological sex Age Health status Pre‐culture processing of the cells	
	**1.3 Initial seeding density**	Initial seeding number per surface area or volume	………………….
	**1.4 Cell density or confluency of cells at the time of CCM collection**	Percentage of surface area covered (2D cultures) Estimated number of cells per volume (suspension cultures) The diameter of the spheroids (3D cultures)	……… % …………………. ………………….
	**1.5 Cell sub‐culturing, population doubling, and passage number**	Passage number Passaging method Recovery time	…………………. …………………. ………………….
	**1.6 Cell viability**	Percentage of viable cells Method of viability assessment	……… % ………………….
**2. EV production medium**	**2.1 Basal medium, glucose, and amino acids**	Name of basal culturing medium Company, and catalog number Additions Salts (name, concentration) Glucose (D/L glucose, concentration) Amino acids (name, concentration)	…………………. …………………. …………….. , …… mM …………….. , …… mM …………….. , …… mM
	**2.2 Antibiotic**	Name Concentration During conditioning or pre‐conditioning or both	……… ……… ……… ……… mM ……… mM ……… mM ………………….
	**2.3 Complex biological supplements**	If the EV production medium contains serum/PL Percentage Producing company, and catalog number of sera Process of PL production, including fibrin depletion methods and pathogen inactivation Concentration of heparin present in the growth medium If the EV production medium contains EV‐depleted serum/PL Indicate whether, how (fold dilution), and with what (buffer, medium) the serum was diluted before EV depletion Method of EV depletion Checking the efficiency of EV depletion Result of checking the efficiency of EV depletion If there are washing steps (before changing to EV depleted medium), write down the details Source, and catalog number of commercial EV‐depleted serum If serum/PL free media is used indicate time duration If using a replacement supplement Percentage Producing company, and catalog number of replacement supplement	…………………. …………………. …………………. …………………. …………………. …………………. …………………. …………………. …………………. …………………. …………………. …………………. ………………….
	**2.4 Cell (pre) conditioning**	Name of factor Company, and catalog number Concentration Conditioning or pre‐conditioning	…………………. ……… ……… ……… ……… mM ……… mM ……… mM ………………….
**3. Culturing conditions**	**3.1 Gas conditioning**	Name (e.g oxygen, nitrogen, etc) Percentage	……… ……… %
	**3.2 2D or 3D cultures: vessels, coatings, matrices**	Type (2D, 3D, or spheroid) Vessels (e.g. flasks or plates) Coating materials 3D support matrix (type, details) Preconditioning with buffers Volume of medium (at the time of CCM harvest for EV preparation) Details of spheroid formation	…………………. …………………. …………………. …………………. …………………. …………………. ………………….
	**3.3 pH**	pH of medium If a pH buffering agent is used indicate its concentration	…………………. ………………….
	**3.4 Physical stimulus**	Type (such as acoustic irradiation, and mechanical forces including shear and stretching of the scaffold) Details Culture temperature	…………………. …………………. …… °C
	**3.5 Replacement of medium**	What percentage of the volume is replaced At what interval (in hours) the medium is changed or replenished Intermediate washing steps (if any) Continuous‐flow feeding (with recirculation or not) Are different media formulations used at various stages of an experiment?	…………………. …………………. …………………. …………………. ………………….
**4. Collection (harvest) of CCM**	**Duration and frequency of CCM collection**	Single harvest: Duration of conditioning (in hours) Multiple harvests: Number of harvests Duration of conditioning (in hours) Whether all or some fraction of medium is collected (i.e., complete or partial medium replacement), specifying the fraction where applicable. How many of the multiple harvests are pooled (if any) Continuous harvest: Duration of collection (in hours) Harvest rate (i.e. volume per unit time)	…………………. …………………. …………………. …………………. ………………. ………………… .…………………
**5. Microbial contaminants of CCM**	**Viruses, bacteria, fungi, and any unwanted microbial components**	Presence of viruses Measurement method When is checked? Outcome Presence of bacteria (e.g. mycoplasma) Measurement method When is checked? Outcome Presence of fungi Measurement method When is checked? Outcome Presence of other unwanted microbial components (e.g. endotoxin) Measurement method When is checked? Outcome	yes no …………………. in producing cells in CCM in both …………………. yes no………………….in producing cells in CCM in both …………………. yes no …………………. in producing cells in CCM in both …………………. yes no…………………. in producing cells in CCM in both ………………….

Abbreviations: CCM, cell culture‐conditioned medium; EV, extracellular vesicle; PL, platelet lysate.

There is hardly any discussion on the mechanisms by which changes in cell culture impact EVs, but these should no longer be overlooked. Cellular processes such as autophagy can affect EV production both directly (Xing et al., 2021; Xu et al., 2018) and indirectly (Moruno et al., 2012; Wang et al., 2021), influencing the number and type of released EVs. Comparing autophagy levels in cultures used for preparing EV batches may explain inter‐experiment / batch differences in EVs. Since hypotonic dialysis can be used to load drugs into EVs (Mehryab et al., 2020), allowing therapeutic agents to cross the membrane (Xie et al., 2021), the osmolarity of the culture medium may affect EV cargo. Given the effects of amino acids on the mammalian target of rapamycin (mTOR) signaling (Jewell et al., 2013) and the connection between mTOR and EV release (Zou et al., 2019), it is reasonable to assume that amino acid concentrations will impact EV production. Finally, oxygen concentration modulates cellular senescence, which may impact EVs, as mentioned in the cellular sub‐culturing section (Seno et al., 2018; Welford & Giaccia, 2011; You et al., 2019).

Of all cell culture supplements, serum and PL are among the most ubiquitous and challenging. They are rich in EVs, their EVs support cell growth and function in ways that cannot easily be achieved with a ‘defined’ medium, and EV depletion from these supplements is time‐consuming and/or inefficient (Lehrich et al., 2021). In addition, they may indirectly influence cells and provide cells with materials that can be re‐packaged into newly produced EVs or incorporated as a bio‐corona (Figure 2d). Hence, more investigations are needed to critically evaluate the consequences of serum/PL removal or EV depletion on each cell type.

In summary, the effects of cell culture parameters on EVs are complex, and our current understanding of these influences is far from complete. A distinction should be made between culturing cells for basic EV research versus therapeutic applications since the latter involved additional considerations around safety and regulation. Missing and vague information about manufacturing and processing parameters can lead to misinterpretations and false conclusions and hampers study reproduction. We therefore emphasize the importance of rigorous reporting of all cell culture parameters to enable researchers to better compare experimental in vitro and in vivo data and pre‐clinical and clinical outcomes. Only with full reporting can we achieve our common goal of comprehensively understanding the biological functions of EVs. We hope that these suggestions (Table 1) will be a useful starting point for further discussions and that they will promote good reporting practices in CCM‐EV research.

## CONFLICT OF INTEREST STATEMENT

The authors declare no conflicts of interest.

## Supporting information

Supporting Information

## References

[jex2115-bib-0001] Agarwal, U. , George, A. , Bhutani, S. , Ghosh‐Choudhary, S. , Maxwell, J. T. , Brown, M. E. , Mehta, Y. , Platt, M. O. , Liang, Y. , Sahoo, S. , & Davis, M. E. (2017). Experimental, systems, and computational approaches to understanding the microRNA‐mediated reparative potential of cardiac progenitor cell‐derived exosomes from pediatric patients. Circulation Research, 120(4), 701–712. 27872050 10.1161/CIRCRESAHA.116.309935 PMC5315680

[jex2115-bib-0002] Antich‐Rosselló, M. , Forteza‐Genestra, M. A. , Monjo, M. , & Ramis, J. M. (2021). Platelet‐derived extracellular vesicles for regenerative medicine. International Journal of Molecular Sciences, 22(16), 8580. 34445286 10.3390/ijms22168580 PMC8395287

[jex2115-bib-0003] Arigony, A. L. , de Oliveira, I. M. , Machado, M. , Bordin, D. L. , Bergter, L. , Prá, D. , & Henriques, J. A. (2013). The influence of micronutrients in cell culture: A reflection on viability and genomic stability. BioMed Research International, 2013, 597282. 23781504 10.1155/2013/597282 PMC3678455

[jex2115-bib-0004] Akbar, N. , Digby, J. E. , Cahill, T. J. , Tavare, A. N. , Corbin, A. L. , Saluja, S. , Dawkins, S. , Edgar, L. , Rawlings, N. , Ziberna, K. , Mcneill, E. , Johnson, E. , Aljabali, A. A. , Dragovic, R. A. , Rohling, M. , Belgard, T. G. , Udalova, I. A. , Greaves, D. R. , Channon, K. M. , … Choudhury, R. P. (2017). Endothelium‐derived extracellular vesicles promote splenic monocyte mobilization in myocardial infarction. JCI Insight, 2(17), e93344. 28878126 10.1172/jci.insight.93344 PMC5621885

[jex2115-bib-0005] Alcolea, J. , Alonso, A. , Moreno‐Izquierdo, M. A. , Degayón, M. A. , Moreno, I. , & Larraga, V. (2016). Serum removal from culture induces growth arrest, ploidy alteration, decrease in infectivity and differential expression of crucial genes in leishmania infantum promastigotes. PLoS ONE, 11(3), e0150172. 26959417 10.1371/journal.pone.0150172 PMC4784933

[jex2115-bib-0006] Alharbi, M. , Lai, A. , Sharma, S. , Kalita‐De Croft, P. , Godbole, N. , Campos, A. , Guanzon, D. , Salas‐Burgos, A. , Carrion, F. , Zuñiga, F. A. , Perrin, L. , He, Y. , Pejovic, T. , Winters, C. , Morgan, T. , Hooper, J. D. , Rice, G. E. , & Salomon, C. (2021). Extracellular vesicle transmission of chemoresistance to ovarian cancer cells is associated with hypoxia‐induced expression of glycolytic pathway proteins, and prediction of epithelial ovarian cancer disease recurrence. Cancers (Basel), 13(14), 3388. 34298602 10.3390/cancers13143388 PMC8305505

[jex2115-bib-0007] Almeria, C. , Weiss, R. , Roy, M. , Tripisciano, C. , Kasper, C. , Weber, V. , & Egger, D. (2019). Hypoxia conditioned mesenchymal stem cell‐derived extracellular vesicles induce increased vascular tube formation in vitro. Frontiers in Bioengineering and Biotechnology, 7, 292. 31709251 10.3389/fbioe.2019.00292 PMC6819375

[jex2115-bib-0008] Almeria, C. , Kreß, S. , Weber, V. , Egger, D. , & Kasper, C. (2022). Heterogeneity of mesenchymal stem cell‐derived extracellular vesicles is highly impacted by the tissue/cell source and culture conditions. Cell & Bioscience, 12(1), 51. 35501833 10.1186/s13578-022-00786-7 PMC9063275

[jex2115-bib-0009] Ambattu, L. A. , Ramesan, S. , Dekiwadia, C. , Hanssen, E. , Li, H. , & Yeo, L. Y. (2020). High frequency acoustic cell stimulation promotes exosome generation regulated by a calcium‐dependent mechanism. Communications Biology, 3(1), 553. 33020585 10.1038/s42003-020-01277-6 PMC7536404

[jex2115-bib-0010] American Type Culture Collection Standards Development Organization Workgroup ASN‐0002 . (2010). Cell line misidentification: The beginning of the end. Nature Reviews Cancer, 10(6), 441–448. 20448633 10.1038/nrc2852

[jex2115-bib-0011] Angelini, F. , Ionta, V. , Rossi, F. , Miraldi, F. , Messina, E. , & Giacomello, A. (2016). Foetal bovine serum‐derived exosomes affect yield and phenotype of human cardiac progenitor cell culture. Bioimpacts, 6(1), 15–24. 27340620 10.15171/bi.2016.03 PMC4916547

[jex2115-bib-0012] Arodin Selenius, L. , Wallenberg Lundgren, M. , Jawad, R. , Danielsson, O. , & Björnstedt, M. (2019). The cell culture medium affects growth, phenotype expression and the response to selenium cytotoxicity in A549 and HepG2 cells. Antioxidants (Basel), 8(5), 130. 31091728 10.3390/antiox8050130 PMC6563005

[jex2115-bib-0013] Aswad, H. , Jalabert, A. , & Rome, S. (2016). Depleting extracellular vesicles from fetal bovine serum alters proliferation and differentiation of skeletal muscle cells in vitro. BMC Biotechnology [Electronic Resource], 16, 32. 27038912 10.1186/s12896-016-0262-0 PMC4818850

[jex2115-bib-0014] Atkin‐Smith, G. K. , Tixeira, R. , Paone, S. , Mathivanan, S. , Collins, C. , Liem, M. , Goodall, K. J. , Ravichandran, K. S. , Hulett, M. D. , & Poon, I. K. H. (2015). A novel mechanism of generating extracellular vesicles during apoptosis via a beads‐on‐a‐string membrane structure. Nature Communications, 6, 7439. 10.1038/ncomms8439 PMC4490561 26074490

[jex2115-bib-0015] Atai, N. A. , Balaj, L. , Van Veen, H. , Breakefield, X. O. , Jarzyna, A. , Van Noorden, C. J. F. , Skog, J. , & Maguire, C. A. (2013). Heparin blocks transfer of extracellular vesicles between donor and recipient cells. Journal of Neuro‐Oncology, 115(3), 343–351. 24002181 10.1007/s11060-013-1235-y PMC3856724

[jex2115-bib-0016] Bai, Q. , Ramirez, J.‐M. , Becker, F. , Pantesco, V. , Lavabre‐Bertrand, T. , Hovatta, O. , Lemaître, J.‐M. , Pellestor, F. , & De Vos, J. (2015). Temporal analysis of genome alterations induced by single‐cell passaging in human embryonic stem cells. Stem Cells and Development, 24(5), 653–662. 25254421 10.1089/scd.2014.0292 PMC4333508

[jex2115-bib-0017] Bai, G. , Matsuba, T. , Niki, T. , & Hattori, T. (2020). Stimulation of THP‐1 macrophages with lps increased the production of osteopontin‐encapsulating exosome. International Journal of Molecular Sciences, 21(22), 8490. 33187327 10.3390/ijms21228490 PMC7696453

[jex2115-bib-0018] Balint, R. , Richardson, S. M. , & Cartmell, S. H. (2015). Low‐density subculture: A technical note on the importance of avoiding cell‐to‐cell contact during mesenchymal stromal cell expansion. Journal of Tissue Engineering and Regenarative Medicine, 9(10), 1200–1203. 10.1002/term.2051 PMC4858810 26153119

[jex2115-bib-0019] Battistelli, M. , & Falcieri, E. (2020). Apoptotic bodies: Particular extracellular vesicles involved in intercellular communication. Biology, 9(1), 21. 31968627 10.3390/biology9010021 PMC7168913

[jex2115-bib-0020] Ban, J.‐J. , Lee, M. , Im, W. , & Kim, M. (2015). Low pH increases the yield of exosome isolation. Biochemical and Biophysical Research Communications, 461(1), 76–79. 25849885 10.1016/j.bbrc.2015.03.172

[jex2115-bib-0021] Barone, W. , Wiebe, M. E. , Leung, J. C. , Hussein, I. T. M. , Keumurian, F. J. , Bouressa, J. , Brussel, A. , Chen, D. , Chong, M. , Dehghani, H. , Gerentes, L. , Gilbert, J. , Gold, D. , Kiss, R. , Kreil, T. R. , Labatut, R. , Li, Y. , Müllberg, J. , Mallet, L. , … Springs, S. L. (2020). Viral contamination in biologic manufacture and implications for emerging therapies. Nature Biotechnology, 38(5), 563–572. 10.1038/s41587-020-0507-2 32341561

[jex2115-bib-0022] Barallon, R. , Bauer, S. R. , Butler, J. , Capes‐Davis, A. , Dirks, W. G. , Elmore, E. , Furtado, M. , Kline, M. C. , Kohara, A. , Los, G. V. , MacLeod, R. A. , Masters, J. R. , Nardone, M. , Nardone, R. M. , Nims, R. W. , Price, J. , Reid, Y. A. , Shewale, J. , Sykes, G. , … Kerrigan, L. (2010). Recommendation of short tandem repeat profiling for authenticating human cell lines, stem cells, and tissues. In Vitro Cellular & Developmental Biology‐Animal, 46(9), 727–732. 20614197 10.1007/s11626-010-9333-z PMC2965362

[jex2115-bib-0023] Baxter, A. A. , Phan, T. K. , Hanssen, E. , Liem, M. , Hulett, M. D. , Mathivanan, S. , & Poon, I. K. H. (2019). Analysis of extracellular vesicles generated from monocytes under conditions of lytic cell death. Scientific Reports, 9(1), 7538. 31101910 10.1038/s41598-019-44021-9 PMC6525174

[jex2115-bib-0024] Becherucci, V. , Piccini, L. , Casamassima, S. , Bisin, S. , Gori, V. , Gentile, F. , Ceccantini, R. , De Rienzo, E. , Bindi, B. , Pavan, P. , Cunial, V. , Allegro, E. , Ermini, S. , Brugnolo, F. , Astori, G. , & Bambi, F. (2018). Human platelet lysate in mesenchymal stromal cell expansion according to a GMP grade protocol: A cell factory experience. Stem Cell Research & Therapy, 9(1), 1–10. 29720245 10.1186/s13287-018-0863-8 PMC5930506

[jex2115-bib-0025] Beer, L. , Zimmermann, M. , Mitterbauer, A. , Ellinger, A. , Gruber, F. , Narzt, M. S. , Zellner, M. , Gyöngyösi, M. , Madlener, S. , Simader, E. , Gabriel, C. , Mildner, M. , & Ankersmit, H. J. (2015). Analysis of the secretome of apoptotic peripheral blood mononuclear cells: Impact of released proteins and exosomes for tissue regeneration. Scientific Reports, 5, 16662. 26567861 10.1038/srep16662 PMC4645175

[jex2115-bib-0026] Beninson, L. A. , & Fleshner, M. (2015). Exosomes in fetal bovine serum dampen primary macrophage IL‐1beta response to lipopolysaccharide (LPS) challenge. Immunology Letters, 163(2), 187–192. 25455591 10.1016/j.imlet.2014.10.019

[jex2115-bib-0027] Bhat, S. , Viswanathan, P. , Chandanala, S. , Prasanna, S. J. , & Seetharam, R. N. (2021). Expansion and characterization of bone marrow derived human mesenchymal stromal cells in serum‐free conditions. Scientific Reports, 11(1), 3403. 33564114 10.1038/s41598-021-83088-1 PMC7873235

[jex2115-bib-0028] Bereiter‐Hahn, J. , Münnich, A. , & Woiteneck, P. (1998). Dependence of energy metabolism on the density of cells in culture. Cell Structure and Function, 23(2), 85–93. 9669036 10.1247/csf.23.85

[jex2115-bib-0029] Beutler, E. , Gelbart, T. , & Kuhl, W. (1990). Interference of heparin with the polymerase chain reaction. Biotechniques, 9(2), 166. 2400599

[jex2115-bib-0030] Biadglegne, F. , Rademacher, P. , De Sulbaran, Y. G. J. , König, B. , Rodloff, A. C. , Zedler, U. , Dorhoi, A. , & Sack, U. (2021). Exosomes in serum‑free cultures of THP‑1 macrophages infected with Mycobacterium tuberculosis. Molecular Medicine Reports, 24(5), 815. 34558650 10.3892/mmr.2021.12455 PMC8477185

[jex2115-bib-0031] Bianchetti, A. , Chinello, C. , Guindani, M. , Braga, S. , Neva, A. , Verardi, R. , Piovani, G. , Pagani, L. , Lisignoli, G. , Magni, F. , Russo, D. , & Almici, C. (2021). A blood bank standardized production of human platelet lysate for mesenchymal stromal cell expansion: proteomic characterization and biological effects. Frontiers in Cell and Developmental Biology, 9, 650490. 34055779 10.3389/fcell.2021.650490 PMC8160451

[jex2115-bib-0032] Bieback, K. , Fernandez‐Muñoz, B. , Pati, S. , & Schäfer, R. (2019). Gaps in the knowledge of human platelet lysate as a cell culture supplement for cell therapy: A joint publication from the AABB and the International Society for Cell & Gene Therapy. Cytotherapy, 21(9), 911–924. 31307904 10.1016/j.jcyt.2019.06.006

[jex2115-bib-0033] Börger, V. , Weiss, D. J. , Anderson, J. D. , Borràs, F. E. , Bussolati, B. , Carter, D. R. F. , Dominici, M. , Falcón‐Pérez, J. M. , Gimona, M. , Hill, A. F. , Hoffman, A. M. , de Kleijn, D. , Levine, B. L. , Lim, R. , Lötvall, J. , Mitsialis, S. A. , Monguió‐Tortajada, M. , Muraca, M. , Nieuwland, R. , … Giebel, B. (2020). International Society for Extracellular Vesicles and International Society for Cell and Gene Therapy statement on extracellular vesicles from mesenchymal stromal cells and other cells: Considerations for potential therapeutic agents to suppress coronavirus disease‐19. Cytotherapy, 22(9), 482–485. 32425691 10.1016/j.jcyt.2020.05.002 PMC7229942

[jex2115-bib-0034] Boulestreau, J. , Maumus, M. , Rozier, P. , Jorgensen, C. , & Noël, D. (2020). Mesenchymal stem cell derived extracellular vesicles in aging. Frontiers in Cell and Developmental Biology, 8, 107. 32154253 10.3389/fcell.2020.00107 PMC7047768

[jex2115-bib-0035] Bost, J. P. , Saher, O. , Hagey, D. , Mamand, D. R. , Liang, X. , Zheng, W. , Corso, G. , Gustafsson, O. , Görgens, A. , Smith, C. E. , Zain, R. , El Andaloussi, S. , & Gupta, D. (2022). Growth media conditions influence the secretion route and release levels of engineered extracellular vesicles. Advanced Healthcare Materials, 11(5), 2101658. 10.1002/adhm.202101658 PMC11469210 34773385

[jex2115-bib-0036] Boussadia, Z. , Lamberti, J. , Mattei, F. , Pizzi, E. , Puglisi, R. , Zanetti, C. , Pasquini, L. , Fratini, F. , Fantozzi, L. , Felicetti, F. , Fecchi, K. , Raggi, C. , Sanchez, M. , D'atri, S. , Carè, A. , Sargiacomo, M. , & Parolini, I. (2018). Acidic microenvironment plays a key role in human melanoma progression through a sustained exosome mediated transfer of clinically relevant metastatic molecules. Journal of Experimental & Clinical Cancer Research, 37(1), 245. 30290833 10.1186/s13046-018-0915-z PMC6173926

[jex2115-bib-0037] Brock, C. K. , Wallin, S. T. , Ruiz, O. E. , Samms, K. M. , Mandal, A. , Sumner, E. A. , & Eisenhoffer, G. T. (2019). Stem cell proliferation is induced by apoptotic bodies from dying cells during epithelial tissue maintenance. Nature Communications, 10(1), 1044. 10.1038/s41467-019-09010-6 PMC6400930 30837472

[jex2115-bib-0038] Burger, D. , Turner, M. , Xiao, F. , Munkonda, M. N. , Akbari, S. , & Burns, K. D. (2017). High glucose increases the formation and pro‐oxidative activity of endothelial microparticles. Diabetologia, 60(9), 1791–1800. 28601907 10.1007/s00125-017-4331-2

[jex2115-bib-0039] Brunner, D. (2010). Serum‐free cell culture: The serum‐free media interactive online database. Altex, 27(1), 53–62. 20390239 10.14573/altex.2010.1.53

[jex2115-bib-0040] Burnouf, T. (2018). Multifaceted regenerative lives of ‘expired’ platelets. ISBT Science Series, 13(3), 323–330.

[jex2115-bib-0041] Buzas, E. I. (2022). Opportunities and challenges in studying the extracellular vesicle corona. Nature Cell Biology, 24(9), 1322–1325. 36042293 10.1038/s41556-022-00983-z

[jex2115-bib-0042] Buzas, E. I. (2023). The roles of extracellular vesicles in the immune system. Nature Reviews Immunology, 23(4), 236–250. 10.1038/s41577-022-00763-8 PMC9361922 35927511

[jex2115-bib-0043] Cavallari, C. , Ranghino, A. , Tapparo, M. , Cedrino, M. , Figliolini, F. , Grange, C. , Giannachi, V. , Garneri, P. , Deregibus, M. C. , Collino, F. , Rispoli, P. , Camussi, G. , & Brizzi, M. F. (2017). Serum‐derived extracellular vesicles (EVs) impact on vascular remodeling and prevent muscle damage in acute hind limb ischemia. Scientific Reports, 7(1), 8180. 28811546 10.1038/s41598-017-08250-0 PMC5557987

[jex2115-bib-0044] Cao, J. , Wang, B. , Tang, T. , Lv, L. , Ding, Z. , Li, Z. , Hu, R. , Wei, Q. , Shen, A. , Fu, Y. , & Liu, B. (2020). Three‐dimensional culture of MSCs produces exosomes with improved yield and enhanced therapeutic efficacy for cisplatin‐induced acute kidney injury. Stem Cell Research Therapy, 11(1), 206. 32460853 10.1186/s13287-020-01719-2 PMC7251891

[jex2115-bib-0045] Capelli, C. , Domenghini, M. , Borleri, G. , Bellavita, P. , Poma, R. , Carobbio, A. , Micò, C. , Rambaldi, A. , Golay, J. , & Introna, M. (2007). Human platelet lysate allows expansion and clinical grade production of mesenchymal stromal cells from small samples of bone marrow aspirates or marrow filter washouts. Bone Marrow Transplantation, 40(8), 785–791. 17680021 10.1038/sj.bmt.1705798

[jex2115-bib-0046] Caruso, S. , & Poon, I. K. H. (2018). Apoptotic cell‐derived extracellular vesicles: More than just debris. Frontiers in Immunology, 9, 1486. 30002658 10.3389/fimmu.2018.01486 PMC6031707

[jex2115-bib-0047] Clabaut, A. , Grare, C. , Léger, T. , Hardouin, P. , & Broux, O. (2015). Variations of secretome profiles according to conditioned medium preparation: The example of human mesenchymal stem cell‐derived adipocytes. Electrophoresis, 36(20), 2587–2593. 26105977 10.1002/elps.201500086

[jex2115-bib-0048] Crescitelli, R. , Lässer, C. , Szabó, T. G. , Kittel, A. , Eldh, M. , Dianzani, I. , Buzás, E. I. , & Lötvall, J. (2013). Distinct RNA profiles in subpopulations of extracellular vesicles: Apoptotic bodies, microvesicles and exosomes. Journal of Extracellular Vesicles, 2(1), 20677. 10.3402/jev.v2i0.20677 PMC3823106 24223256

[jex2115-bib-0049] Charoenviriyakul, C. , Takahashi, Y. , Morishita, M. , Matsumoto, A. , Nishikawa, M. , & Takakura, Y. (2017). Cell type‐specific and common characteristics of exosomes derived from mouse cell lines: Yield, physicochemical properties, and pharmacokinetics. European Journal of Pharmaceutical Sciences, 96, 316–322. 27720897 10.1016/j.ejps.2016.10.009

[jex2115-bib-0050] Cheng, Y. , Zeng, Q. , Han, Q. , & Xia, W. (2019). Effect of pH, temperature and freezing‐thawing on quantity changes and cellular uptake of exosomes. Protein Cell, 10(4), 295–299. 29616487 10.1007/s13238-018-0529-4 PMC6418301

[jex2115-bib-0051] Choudhery, M. S. , Badowski, M. , Muise, A. , Pierce, J. , & Harris, D. T. (2014). Donor age negatively impacts adipose tissue‐derived mesenchymal stem cell expansion and differentiation. Journal of Translational Medicine, 12, 8. 24397850 10.1186/1479-5876-12-8 PMC3895760

[jex2115-bib-0052] Clayton, A. , Boilard, E. , Buzas, E. I. , Cheng, L. , Falcón‐Perez, J. M. , Gardiner, C. , Gustafson, D. , Gualerzi, A. , Hendrix, A. , Hoffman, A. , Jones, J. , Lässer, C. , Lawson, C. , Lenassi, M. , Nazarenko, I. , O'driscoll, L. , Pink, R. , Siljander, R.‐M. , Soekmadji, C. , … Nieuwland, R. (2019). Considerations towards a roadmap for collection, handling and storage of blood extracellular vesicles. Journal of Extracellular Vesicles, 8(1), 1647027–1647027. 31489143 10.1080/20013078.2019.1647027 PMC6711123

[jex2115-bib-0053] Coecke, S. , Balls, M. , Bowe, G. , Davis, J. , Gstraunthaler, G. , Hartung, T. , Hay, R. , Merten, O.‐W. , Price, A. , Schechtman, L. , Stacey, G. , & Stokes, W. (2005). Guidance on good cell culture practice. A report of the second ECVAM task force on good cell culture practice. Alternatives to Laboratory Animals, 33(3), 261–287. 16180980 10.1177/026119290503300313

[jex2115-bib-0054] Cohen, S. , Samadikuchaksaraei, A. , Polak, J. M. , & Bishop, A. E. (2006). Antibiotics reduce the growth rate and differentiation of embryonic stem cell cultures. Tissue Engineering, 12(7), 2025–2030. 16889530 10.1089/ten.2006.12.2025

[jex2115-bib-0055] Commission, E.P., E.D.f.t.Q.o . (2010). Medicines, and Healthcare, European pharmacopoeia., Vol. 1. Council of Europe.

[jex2115-bib-0056] Cossetti, C. , Iraci, N. , Mercer, T. R. , Leonardi, T. , Alpi, E. , Drago, D. , Alfaro‐Cervello, C. , Saini, H. K. , Davis, M. P. , Schaeffer, J. , Vega, B. , Stefanini, M. , Zhao, C. , Muller, W. , Garcia‐Verdugo, J. M. , Mathivanan, S. , Bachi, A. , Enright, A. J. , Mattick, J. S. , & Pluchino, S. (2014). Extracellular vesicles from neural stem cells transfer IFN‐gamma via Ifngr1 to activate Stat1 signaling in target cells. Molecular Cell, 56(2), 193–204. 25242146 10.1016/j.molcel.2014.08.020 PMC4578249

[jex2115-bib-0057] Costa, L. A. , Eiro, N. , Fraile, M. , Gonzalez, L. O. , Saá, J. , Garcia‐Portabella, P. , Vega, B. , Schneider, J. , & Vizoso, F. J. (2021). Functional heterogeneity of mesenchymal stem cells from natural niches to culture conditions: Implications for further clinical uses. Cellular and Molecular Life Sciences, 78(2), 447–467. 32699947 10.1007/s00018-020-03600-0 PMC7375036

[jex2115-bib-0058] Cronemberger‐Andrade, A. , Xander, P. , Soares, R. P. , Pessoa, N. L. , Campos, M. A. , Ellis, C. C. , Grajeda, B. , Ofir‐Birin, Y. , Almeida, I. C. , Regev‐Rudzki, N. , & Torrecilhas, A. C. (2020). Trypanosoma cruzi‐infected human macrophages shed proinflammatory extracellular vesicles that enhance host‐cell invasion via toll‐like receptor 2. Frontiers in Cellular and Infection Microbiology, 10, 99. 32266161 10.3389/fcimb.2020.00099 PMC7098991

[jex2115-bib-0059] Dai, J.‐M. , Yu, M.‐X. , Shen, Z.‐Y. , Guo, C.‐Y. , Zhuang, S.‐Q. , & Qiu, X.‐S. (2015). Leucine promotes proliferation and differentiation of primary preterm rat satellite cells in part through mTORC1 signaling pathway. Nutrients, 7(5), 3387–3400. 26007333 10.3390/nu7053387 PMC4446757

[jex2115-bib-0060] Davidson, S. M. , Riquelme, J. A. , Zheng, Y. , Vicencio, J. M. , Lavandero, S. , & Yellon, D. M. (2018a). Endothelial cells release cardioprotective exosomes that may contribute to ischaemic preconditioning. Scientific Reports, 8(1), 1–9. 30367147 10.1038/s41598-018-34357-z PMC6203728

[jex2115-bib-0061] Davidson, S. M. , Riquelme, J. A. , Takov, K. , Vicencio, J. M. , Boi‐Doku, C. , Khoo, V. , Doreth, C. , Radenkovic, D. , Lavandero, S. , & Yellon, D. M. (2018b). Cardioprotection mediated by exosomes is impaired in the setting of type II diabetes but can be rescued by the use of non‐diabetic exosomes in vitro. Journal of Cellular and Molecular Medicine, 22(1), 141–151. 28840975 10.1111/jcmm.13302 PMC5742744

[jex2115-bib-0062] De Almeida Fuzeta, M. , Bernardes, N. , Oliveira, F. D. , Costa, A. C. , Fernandes‐Platzgummer, A. , Farinha, J. P. , Rodrigues, C. A. V. , Jung, S. , Tseng, R.‐J. , Milligan, W. , Lee, B. , Castanho, M. A. R. B. , Gaspar, D. , Cabral, J. M. S. , & Da Silva, C. L. (2020). Scalable production of human mesenchymal stromal cell‐derived extracellular vesicles under serum‐/xeno‐free conditions in a microcarrier‐based bioreactor culture system. Frontiers in Cell and Developmental Biology, 8, 553444. 33224943 10.3389/fcell.2020.553444 PMC7669752

[jex2115-bib-0063] De Jong, O. G. , Verhaar, M. C. , Chen, Y. , Vader, P. , Gremmels, H. , Posthuma, G. , Schiffelers, R. M. , Gucek, M. , & Van Balkom, B. W. M. (2012). Cellular stress conditions are reflected in the protein and RNA content of endothelial cell‐derived exosomes. Journal of Extracellular Vesicles, 1, 18396. 10.3402/jev.v1i0.18396 PMC3760650 24009886

[jex2115-bib-0064] Dieudé, M. , Bell, C. , Turgeon, J. , Beillevaire, D. , Pomerleau, L. , Yang, B. , Hamelin, K. , Qi, S. , Pallet, N. , Béland, C. , Dhahri, W. , Cailhier, J.‐F. , Rousseau, M. , Duchez, A.‐C. , Lévesque, T. , Lau, A. , Rondeau, C. , Gingras, D. , Muruve, D. , … Hébert, M.‐J. (2015). The 20S proteasome core, active within apoptotic exosome‐like vesicles, induces autoantibody production and accelerates rejection. Science Translational Medicine, 7(318), 318ra200. 10.1126/scitranslmed.aac9816 26676607

[jex2115-bib-0065] Dominici, M. , Le Blanc, K. , Mueller, I. , Slaper‐Cortenbach, I. , Marini, F. C. , Krause, D. S. , Deans, R. J. , Keating, A. , Prockop, D. J. , & Horwitz, E. M. (2006). Minimal criteria for defining multipotent mesenchymal stromal cells. The International Society for Cellular Therapy position statement. Cytotherapy, 8(4), 315–317. 16923606 10.1080/14653240600855905

[jex2115-bib-0066] Dorronsoro, A. , Santiago, F. E. , Grassi, D. , Zhang, T. , Lai, R. C. , Mcgowan, S. J. , Angelini, L. , Lavasani, M. , Corbo, L. , Lu, A. , Brooks, R. W. , Garcia‐Contreras, M. , Stolz, D. B. , Amelio, A. , Boregowda, S. V. , Fallahi, M. , Reich, A. , Ricordi, C. , Phinney, D. G. , … Robbins, D. (2021). Mesenchymal stem cell‐derived extracellular vesicles reduce senescence and extend health span in mouse models of aging. Aging Cell, 20(4), e13337. 33728821 10.1111/acel.13337 PMC8045949

[jex2115-bib-0067] Dougherty, J. A. , Patel, N. , Kumar, N. , Rao, S. G. , Angelos, M. G. , Singh, H. , Cai, C. , & Khan, M. (2020). Human cardiac progenitor cells enhance exosome release and promote angiogenesis under physoxia. Frontiers in Cell and Developmental Biology, 8, 130. 32211408 10.3389/fcell.2020.00130 PMC7068154

[jex2115-bib-0068] Driedonks, T. A. P. , Nijen Twilhaar, M. K. , & Nolte‐'t Hoen, E. N. M. (2019). Technical approaches to reduce interference of Fetal calf serum derived RNA in the analysis of extracellular vesicle RNA from cultured cells. Journal of Extracellular Vesicles, 8(1), 1552059. 30559953 10.1080/20013078.2018.1552059 PMC6292350

[jex2115-bib-0069] Dutta, S. , Lai, A. , Scholz‐Romero, K. , Shiddiky, M. J. A. , Yamauchi, Y. , Mishra, J. S. , Rice, G. E. , Hyett, J. , Kumar, S. , & Salomon, C. (2020). Hypoxia‐induced small extracellular vesicle proteins regulate proinflammatory cytokines and systemic blood pressure in pregnant rats. Clinical Science (London, England: 1979), 134(6), 593–607. 32129439 10.1042/CS20191155 PMC7346733

[jex2115-bib-0070] Eichholz, K. F. , Woods, I. , Riffault, M. , Johnson, G. P. , Corrigan, M. , Lowry, M. C. , Shen, N. , Labour, M.‐N. , Wynne, K. , O'driscoll, L. , & Hoey, D. A. (2020). Human bone marrow stem/stromal cell osteogenesis is regulated via mechanically activated osteocyte‐derived extracellular vesicles. Stem Cells Translational Medicine, 9(11), 1431–1447. 32672416 10.1002/sctm.19-0405 PMC7581449

[jex2115-bib-0071] Eitan, E. , Zhang, S. , Witwer, K. W. , & Mattson, M. P. (2015). Extracellular vesicle‐depleted fetal bovine and human sera have reduced capacity to support cell growth. Journal of Extracellular Vesicles, 4, 26373. 25819213 10.3402/jev.v4.26373 PMC4376846

[jex2115-bib-0072] Elahi, F. M. , Farwell, D. G. , Nolta, J. A. , & Anderson, J. D. (2020). Preclinical translation of exosomes derived from mesenchymal stem/stromal cells. Stem Cells, 38(1), 15–21. 31381842 10.1002/stem.3061 PMC7004029

[jex2115-bib-0073] Erdbrügger, U. , Blijdorp, C. J. , Bijnsdorp, I. V. , Borràs, F. E. , Burger, D. , Bussolati, B. , Byrd, J. B. , Clayton, A. , Dear, J. W. , Falcón‐Pérez, J. M. , Grange, C. , Hill, A. F. , Holthöfer, H. , Hoorn, E. J. , Jenster, G. , Jimenez, C. R. , Junker, K. , Klein, J. , Knepper, M. A. , … Martens‐Uzunova, E. S. (2021). Urinary extracellular vesicles: A position paper by the Urine Task Force of the International Society for Extracellular Vesicles. Journal of Extracellular Vesicles, 10(7), e12093. 34035881 10.1002/jev2.12093 PMC8138533

[jex2115-bib-0074] Emam, S. E. , Ando, H. , Abu Lila, A. S. , Shimizu, T. , Ukawa, M. , Okuhira, K. , Ishima, Y. , Mahdy, M. A. , Ghazy, F.‐E. S. , & Ishida, T. (2018). A novel strategy to increase the yield of exosomes (extracellular vesicles) for an expansion of basic research. Biological & Pharmaceutical Bulletin, 41(5), 733–742. 29709910 10.1248/bpb.b17-00919

[jex2115-bib-0075] Fafián‐Labora, J. , Lesende‐Rodriguez, I. , Fernández‐Pernas, P. , Sangiao‐Alvarellos, S. , Monserrat, L. , Arntz, O. J. , Van De Loo, F. A. J. , Mateos, J. , & Arufe, M. C. (2017). Effect of age on pro‐inflammatory miRNAs contained in mesenchymal stem cell‐derived extracellular vesicles. Scientific Reports, 7, 43923. 28262816 10.1038/srep43923 PMC5338265

[jex2115-bib-0076] Fan, S.‐J. , Kroeger, B. , Marie, P. , Bridges, E. M. , Mason, J. D. , Mccormick, K. , Zois, C. E. , Sheldon, H. , Khalid Alham, N. , Johnson, E. , Ellis, M. , Stefana, M. I. , Mendes, C. C. , Wainwright, S. M. , Cunningham, C. , Hamdy, F. C. , Morris, J. F. , Harris, A. L. , Wilson, C. , & Goberdhan, D. C. (2020). Glutamine deprivation alters the origin and function of cancer cell exosomes. Embo Journal, 39(16), e103009. 32720716 10.15252/embj.2019103009 PMC7429491

[jex2115-bib-0077] Faruqu, F. N. , Liam‐Or, R. , Zhou, S. , Nip, R. , & Al‐Jamal, K. T. (2021). Defined serum‐free three‐dimensional culture of umbilical cord‐derived mesenchymal stem cells yields exosomes that promote fibroblast proliferation and migration in vitro. The FASEB Journal, 35(1), e21206. 33368666 10.1096/fj.202001768RR PMC7986687

[jex2115-bib-0078] Figueroa‐Valdés, A. I. , De La Fuente, C. , Hidalgo, Y. , Vega‐Letter, A. M. , Tapia‐Limonchi, R. , Khoury, M. , & Alcayaga‐Miranda, F. (2021). A chemically defined, xeno‐ and blood‐free culture medium sustains increased production of small extracellular vesicles from mesenchymal stem cells. Frontiers in Bioengineering and Biotechnology, 9, 619930. 34124014 10.3389/fbioe.2021.619930 PMC8187876

[jex2115-bib-0079] Fisher, H. W. , Puck, T. T. , & Sato, G. (1958). Molecular growth requirements of single mammalian cells: The action of fetuin in promoting cell attachment to glass. PNAS, 44(1), 4–10. 16590146 10.1073/pnas.44.1.4 PMC335353

[jex2115-bib-0080] Fliefel, R. , Popov, C. , Tröltzsch, M. , Kühnisch, J. , Ehrenfeld, M. , & Otto, S. (2016). Mesenchymal stem cell proliferation and mineralization but not osteogenic differentiation are strongly affected by extracellular pH. Journal of Cranio‐Maxillofacial Surgery, 44(6), 715–724. 27085985 10.1016/j.jcms.2016.03.003

[jex2115-bib-0081] Flores‐Bellver, M. , Mighty, J. , Aparicio‐Domingo, S. , Li, K. V. , Shi, C. , Zhou, J. , Cobb, H. , Mcgrath, P. , Michelis, G. , Lenhart, P. , Bilousova, G. , Heissel, S. , Rudy, M. J. , Coughlan, C. , Goodspeed, A. E. , Becerra, S. P. , Redenti, S. , & Canto‐Soler, M. V. (2021). Extracellular vesicles released by human retinal pigment epithelium mediate increased polarised secretion of drusen proteins in response to AMD stressors. Journal of Extracellular Vesicles, 10(13), e12165. 34750957 10.1002/jev2.12165 PMC8575963

[jex2115-bib-0082] Frigerio, R. , Musicò, A. , Brucale, M. , Ridolfi, A. , Galbiati, S. , Vago, R. , Bergamaschi, G. , Ferretti, A. M. , Chiari, M. , Valle, F. , Gori, A. , & Cretich, M. (2021). Extracellular vesicles analysis in the COVID‐19 era: Insights on serum inactivation protocols towards downstream isolation and analysis. Cells, 10(3), 544. 33806297 10.3390/cells10030544 PMC8001372

[jex2115-bib-0083] Fukuta, T. , Nishikawa, A. , & Kogure, K. (2020). Low level electricity increases the secretion of extracellular vesicles from cultured cells. Biochemistry and Biophysics Report, 21, 100713. 10.1016/j.bbrep.2019.100713 PMC6889636 31828227

[jex2115-bib-0084] Gaurivaud, P. , Ganter, S. , Villard, A. , Manso‐Silvan, L. , Chevret, D. , Boulé, C. , Monnet, V. , & Tardy, F. (2018). Mycoplasmas are no exception to extracellular vesicles release: Revisiting old concepts. PLoS ONE, 13(11), e0208160. 30485365 10.1371/journal.pone.0208160 PMC6261642

[jex2115-bib-0085] Garcia, N. A. , Ontoria‐Oviedo, I. , González‐King, H. , Diez‐Juan, A. , & Sepúlveda, P. (2015). Glucose starvation in cardiomyocytes enhances exosome secretion and promotes angiogenesis in endothelial cells. PLoS ONE, 10(9), e0138849. 26393803 10.1371/journal.pone.0138849 PMC4578916

[jex2115-bib-0086] Gimona, M. , Brizzi, M. F. , Choo, A. B. H. , Dominici, M. , Davidson, S. M. , Grillari, J. , Hermann, D. M. , Hill, A. F. , De Kleijn, D. , Lai, R. C. , Lai, C. P. , Lim, R. , Monguió‐Tortajada, M. , Muraca, M. , Ochiya, T. , Ortiz, L. A. , Toh, W. S. , Yi, Y. W. , Witwer, K. W. , … Lim, S. K. (2021). Critical considerations for the development of potency tests for therapeutic applications of mesenchymal stromal cell‐derived small extracellular vesicles. Cytotherapy, 23(5), 373–380. 33934807 10.1016/j.jcyt.2021.01.001

[jex2115-bib-0087] Gottipamula, S. , Sharma, A. , Krishnamurthy, S. , Majumdar, A. S. , & Seetharam, R. N. (2012). Human platelet lysate is an alternative to fetal bovine serum for large‐scale expansion of bone marrow‐derived mesenchymal stromal cells. Biotechnology Letters, 34(7), 1367–1374. 22476583 10.1007/s10529-012-0893-8

[jex2115-bib-0088] Grangier, A. , Branchu, J. , Volatron, J. , Piffoux, M. , Gazeau, F. , Wilhelm, C. , & Silva, A. K. A. (2021). Technological advances towards extracellular vesicles mass production. Advanced Drug Delivery Reviews, 176, 113843. 34147532 10.1016/j.addr.2021.113843

[jex2115-bib-0089] Griffiths, S. , Baraniak, R. , Copland, I. B. , Nerem, R. M. , & Mcdevitt, T. C. (2013). Human platelet lysate stimulates high‐passage and senescent human multipotent mesenchymal stromal cell growth and rejuvenation in vitro. Cytotherapy, 15(12), 1469–1483. 23981539 10.1016/j.jcyt.2013.05.020

[jex2115-bib-0090] Gregory, C. D. , & Dransfield, I. (2018). Apoptotic tumor cell‐derived extracellular vesicles as important regulators of the onco‐regenerative niche. Frontiers in Immunology, 9, 1111. 29875772 10.3389/fimmu.2018.01111 PMC5974173

[jex2115-bib-0091] Gruber, H. E. , Somayaji, S. , Riley, F. , Hoelscher, G. L. , Norton, H. J. , Ingram, J. , & Hanley, E. N. Jr (2012). Human adipose‐derived mesenchymal stem cells: Serial passaging, doubling time and cell senescence. Biotechnic & Histochemistry, 87(4), 303–311. 22250760 10.3109/10520295.2011.649785

[jex2115-bib-0092] Galluzzi, L. , Vitale, I. , Aaronson, S. A. , Abrams, J. M. , Adam, D. , Agostinis, P. , Alnemri, E. S. , Altucci, L. , Amelio, I. , Andrews, D. W. , Annicchiarico‐Petruzzelli, M. , Antonov, A. V. , Arama, E. , Baehrecke, E. H. , Barlev, N. A. , Bazan, N. G. , Bernassola, F. , Bertrand, M. J. M. , Bianchi, K. , … Kroemer, G. (2018). Molecular mechanisms of cell death: Recommendations of the nomenclature committee on cell death 2018. Cell Death & Differentiation, 25(3), 486–541. 29362479 10.1038/s41418-017-0012-4 PMC5864239

[jex2115-bib-0093] Gao, W. , Liang, T. , He, R. , Ren, J. , Yao, H. , Wang, K. , Zhu, L. , & Xu, Y. (2020). Exosomes from 3D culture of marrow stem cells enhances endothelial cell proliferation, migration, and angiogenesis via activation of the HMGB1/AKT pathway. Stem Cell Research, 50, 102122. 33316600 10.1016/j.scr.2020.102122

[jex2115-bib-0094] Garitaonandia, I. , Amir, H. , Boscolo, F. S. , Wambua, G. K. , Schultheisz, H. L. , Sabatini, K. , Morey, R. , Waltz, S. , Wang, Y.‐C. , Tran, H. , Leonardo, T. R. , Nazor, K. , Slavin, I. , Lynch, C. , Li, Y. , Coleman, R. , Gallego Romero, I. , Altun, G. , Reynolds, D. , … Laurent, L. C. (2015). Increased risk of genetic and epigenetic instability in human embryonic stem cells associated with specific culture conditions. PLoS ONE, 10(2), e0118307. 25714340 10.1371/journal.pone.0118307 PMC4340884

[jex2115-bib-0095] Gstraunthaler, G. (2003). Alternatives to the use of fetal bovine serum: Serum‐free cell culture. Altex, 20(4), 275–281. 14671707

[jex2115-bib-0096] Gu, H. , Liu, Z. , Li, Y. , Xie, Y. , Yao, J. , Zhu, Y. , Xu, J. , Dai, Q. , Zhong, C. , Zhu, H. , Ding, S. , & Zhou, L. (2018). Serum‐Derived Extracellular Vesicles Protect Against Acute Myocardial Infarction by Regulating miR‐21/PDCD4 Signaling Pathway. Frontiers in Physiology, 9, 348. 29674977 10.3389/fphys.2018.00348 PMC5895646

[jex2115-bib-0097] Gudbergsson, J. M. , Johnsen, K. B. , Skov, M. N. , & Duroux, M. (2016). Systematic review of factors influencing extracellular vesicle yield from cell cultures. Cytotechnology, 68(4), 579–592. 26433593 10.1007/s10616-015-9913-6 PMC4960200

[jex2115-bib-0098] Guerreiro, E. M. , Vestad, B. , Steffensen, L. A. , Aass, H. C. D. , Saeed, M. , Øvstebø, R. , Costea, D. E. , Galtung, H. K. , & Søland, T. M. (2018). Efficient extracellular vesicle isolation by combining cell media modifications, ultrafiltration, and size‐exclusion chromatography. PLoS ONE, 13(9), e0204276. 30260987 10.1371/journal.pone.0204276 PMC6160036

[jex2115-bib-0099] Gurunathan, S. , Kang, M.‐H. , & Kim, J.‐H. (2021). A comprehensive review on factors influences biogenesis, functions, therapeutic and clinical implications of exosomes. International Journal of Nanomedicine, 16, 1281–1312. 33628021 10.2147/IJN.S291956 PMC7898217

[jex2115-bib-0100] Guiotto, M. , Raffoul, W. , Hart, A. M. , Riehle, M. O. , & Di Summa, G. (2020). Human platelet lysate to substitute fetal bovine serum in hMSC expansion for translational applications: A systematic review. Journal of Translational Medicine, 18(1), 351. 32933520 10.1186/s12967-020-02489-4 PMC7493356

[jex2115-bib-0101] Guo, S. , Debbi, L. , Zohar, B. , Samuel, R. , Arzi, R. S. , Fried, A. I. , Carmon, T. , Shevach, D. , Redenski, I. , Schlachet, I. , Sosnik, A. , & Levenberg, S. (2021). Stimulating extracellular vesicles production from engineered tissues by mechanical forces. Nano Letters, 21(6), 2497–2504. 33709717 10.1021/acs.nanolett.0c04834

[jex2115-bib-0102] György, B. , Szabó, T. G. , Pásztói, M. , Pál, Z. , Misják, P. , Aradi, B. , László, V. , Pállinger, É. , Pap, E. , Kittel, Á. , Nagy, G. , Falus, A. , & Buzás, E. I. (2011). Membrane vesicles, current state‐of‐the‐art: Emerging role of extracellular vesicles. Cellular and Molecular Life Sciences, 68(16), 2667–2688. 21560073 10.1007/s00018-011-0689-3 PMC3142546

[jex2115-bib-0103] Haraszti, R. A. , Miller, R. , Stoppato, M. , Sere, Y. Y. , Coles, A. , Didiot, M.‐C. , Wollacott, R. , Sapp, E. , Dubuke, M. L. , Li, X. , Shaffer, S. A. , Difiglia, M. , Wang, Y. , Aronin, N. , & Khvorova, A. (2018). Exosomes produced from 3D cultures of MSCs by tangential flow filtration show higher yield and improved activity. Molecular Therapy, 26(12), 2838–2847. 30341012 10.1016/j.ymthe.2018.09.015 PMC6277553

[jex2115-bib-0104] Hayes, O. , Ramos, B. , Rodríguez, L. L. , Aguilar, A. , Badía, T. , & Castro, F. O. (2005). Cell confluency is as efficient as serum starvation for inducing arrest in the G0/G1 phase of the cell cycle in granulosa and fibroblast cells of cattle. Animal Reproduction Science, 87(3‐4), 181–192. 15911169 10.1016/j.anireprosci.2004.11.011

[jex2115-bib-0105] Hawkes, W. (2015). Fetal bovine serum: Geographic origin and regulatory relevance of viral contamination. Bioresources and Bioprocessing, 2(1), 34.

[jex2115-bib-0106] Hemeda, H. , Giebel, B. , & Wagner, W. (2014). Evaluation of human platelet lysate versus fetal bovine serum for culture of mesenchymal stromal cells. Cytotherapy, 16(2), 170–180. 24438898 10.1016/j.jcyt.2013.11.004

[jex2115-bib-0107] Henschler, R. , Gabriel, C. , Schallmoser, K. , Burnouf, T. , & Koh, M. B. C. (2019). Human platelet lysate current standards and future developments. Transfusion, 59(4), 1407–1413. 30741431 10.1111/trf.15174

[jex2115-bib-0108] Hergenreider, E. , Heydt, S. , Tréguer, K. , Boettger, T. , Horrevoets, A. J. G. , Zeiher, A. M. , Scheffer, M. P. , Frangakis, A. S. , Yin, X. , Mayr, M. , Braun, T. , Urbich, C. , Boon, R. A. , & Dimmeler, S. (2012). Atheroprotective communication between endothelial cells and smooth muscle cells through miRNAs. Nature Cell Biology, 14(3), 249–256. 22327366 10.1038/ncb2441

[jex2115-bib-0109] Hill, A. F. , Pegtel, D. M. , Lambertz, U. , Leonardi, T. , O'driscoll, L. , Pluchino, S. , Ter‐Ovanesyan, D. , & Nolte‐‘T Hoen, E. N. M. (2013). ISEV position paper: Extracellular vesicle RNA analysis and bioinformatics. Journal of Extracellular Vesicles, 2, 22859. 10.3402/jev.v2i0.22859 PMC3873759 24376909

[jex2115-bib-0110] Ho, J. H. , Chen, Y.‐F. , Ma, W.‐H. , Tseng, T.‐C. , Chen, M.‐H. , & Lee, O. K. (2011). Cell contact accelerates replicative senescence of human mesenchymal stem cells independent of telomere shortening and p53 activation: Roles of Ras and oxidative stress. Cell Transplantation, 20(8), 1209–1220. 21176396 10.3727/096368910X546562

[jex2115-bib-0111] Hosseinkhani, B. , Van Den Akker, N. , D'haen, J. , Gagliardi, M. , Struys, T. , Lambrichts, I. , Waltenberger, J. , Nelissen, I. , Hooyberghs, J. , Molin, D. G. M. , & Michiels, L. (2017). Direct detection of nano‐scale extracellular vesicles derived from inflammation‐triggered endothelial cells using surface plasmon resonance. Nanomedicine, 13(5), 1663–1671. 28366819 10.1016/j.nano.2017.03.010

[jex2115-bib-0112] Hosseinkhani, B. , Van Den Akker, N. M. S. , Molin, D. G. M. , & Michiels, L. (2020). (Sub)populations of extracellular vesicles released by TNF‐α ‐triggered human endothelial cells promote vascular inflammation and monocyte migration. Journal of Extracellular Vesicles, 9(1), 1801153. 32944190 10.1080/20013078.2020.1801153 PMC7480596

[jex2115-bib-0113] Huang, Y. , Li, R. , Zhang, L. , Chen, Y. , Dong, W. , Zhao, X. , Yang, H. , Zhang, S. , Xie, Z. , Ye, Z. , Wang, W. , Li, C. , Li, Z. , Liu, S. , Dong, Z. , Yu, X. , & Liang, X. (2020). Extracellular vesicles from high glucose‐treated podocytes induce apoptosis of proximal tubular epithelial cells. Frontiers in Physiology, 11, 579296. 33224036 10.3389/fphys.2020.579296 PMC7667287

[jex2115-bib-0114] Hurwitz, S. N. , Conlon, M. M. , Rider, M. A. , Brownstein, N. C. , & Meckes, D. G. (2016). Nanoparticle analysis sheds budding insights into genetic drivers of extracellular vesicle biogenesis. Journal of Extracellular Vesicles, 5(1), 31295. 27421995 10.3402/jev.v5.31295 PMC4947197

[jex2115-bib-0115] Jacobs, K. , Zambelli, F. , Mertzanidou, A. , Smolders, I. , Geens, M. , Nguyen, H. T. , Barbé, L. , Sermon, K. , & Spits, C. (2016). Higher‐density culture in human embryonic stem cells results in DNA damage and genome instability. Stem Cell Reports, 6(3), 330–341. 26923824 10.1016/j.stemcr.2016.01.015 PMC4788786

[jex2115-bib-0116] Jangamreddy, J. R. , Haagdorens, M. K. C. , Mirazul Islam, M. , Lewis, P. , Samanta, A. , Fagerholm, P. , Liszka, A. , Ljunggren, M. K. , Buznyk, O. , Alarcon, E. I. , Zakaria, N. , Meek, K. M. , & Griffith, M. (2018). Short peptide analogs as alternatives to collagen in pro‐regenerative corneal implants. Acta Biomaterialia, 69, 120–130. 29355715 10.1016/j.actbio.2018.01.011 PMC5842042

[jex2115-bib-0117] Jarmalavičiūtė, A. , Tunaitis, V. , Pivoraitė, U. , Venalis, A. , & Pivoriūnas, A. (2015). Exosomes from dental pulp stem cells rescue human dopaminergic neurons from 6‐hydroxy‐dopamine‐induced apoptosis. Cytotherapy, 17(7), 932–939. 25981557 10.1016/j.jcyt.2014.07.013

[jex2115-bib-0118] Jewell, J. L. , Russell, R. C. , & Guan, K.‐L. (2013). Amino acid signalling upstream of mTOR. Nature Reviews Molecular Cell Biology, 14(3), 133–139. 23361334 10.1038/nrm3522 PMC3988467

[jex2115-bib-0119] Johansson, L. , Klinth, J. , Holmqvist, O. , & Ohlson, S. (2003). Platelet lysate: A replacement for fetal bovine serum in animal cell culture? Cytotechnology, 42(2), 67–74. 19002929 10.1023/B:CYTO.0000009820.72920.cf PMC3449796

[jex2115-bib-0120] Juhl, M. , Tratwal, J. , Follin, B. , Søndergaard, R. H. , Kirchhoff, M. , Ekblond, A. , Kastrup, J. , & Haack‐Sørensen, M. (2016). Comparison of clinical grade human platelet lysates for cultivation of mesenchymal stromal cells from bone marrow and adipose tissue. Scandinavian Journal of Clinical and Laboratory Investigation, 76(2), 93–104. 26878874 10.3109/00365513.2015.1099723

[jex2115-bib-0121] Kakarla, R. , Hur, J. , Kim, Y. J. , Kim, J. , & Chwae, Y. J. (2020). Apoptotic cell‐derived exosomes: Messages from dying cells. Experimental & Molecular Medicine, 52(1), 1–6. 31915368 10.1038/s12276-019-0362-8 PMC7000698

[jex2115-bib-0122] Kang, T. , Jones, T. M. , Naddell, C. , Bacanamwo, M. , Calvert, J. W. , Thompson, W. E. , Bond, V. C. , Chen, Y. E. , & Liu, D. (2016). Adipose‐derived stem cells induce angiogenesis via microvesicle transport of miRNA‐31. Stem Cells Translational Medicine, 5(4), 440–450. 26933040 10.5966/sctm.2015-0177 PMC4798737

[jex2115-bib-0123] Kawakami, T. , Kawamura, K. , Fujimori, K. , Koike, A. , & Amano, F. (2016). Influence of the culture medium on the production of nitric oxide and expression of inducible nitric oxide synthase by activated macrophages in vitro. Biochemistry and Biophysics Report, 5, 328–334. 10.1016/j.bbrep.2016.01.006 PMC5600421 28955839

[jex2115-bib-0124] Katsara, O. , Mahaira, L. G. , Iliopoulou, E. G. , Moustaki, A. , Antsaklis, A. , Loutradis, D. , Stefanidis, K. , Baxevanis, C. N. , Papamichail, M. , & Perez, S. A. (2011). Effects of donor age, gender, and in vitro cellular aging on the phenotypic, functional, and molecular characteristics of mouse bone marrow‐derived mesenchymal stem cells. Stem Cells and Development, 20(9), 1549–1561. 21204633 10.1089/scd.2010.0280

[jex2115-bib-0125] Kassem, M. , Ankersen, L. , Eriksen, E. F. , Clark, B. F. C. , & Rattan, S. I. S. (1997). Demonstration of cellular aging and senescence in serially passaged long‐term cultures of human trabecular osteoblasts. Osteoporosis International, 7(6), 514–524. 9604046 10.1007/BF02652556

[jex2115-bib-0126] Kerris, E. W. J. , Hoptay, C. , Calderon, T. , & Freishtat, R. J. (2020). Platelets and platelet extracellular vesicles in hemostasis and sepsis. Journal of Investigative Medicine, 68(4), 813–820. 31843956 10.1136/jim-2019-001195

[jex2115-bib-0127] Kim, N. (2021). pH variation impacts molecular pathways associated with somatic cell reprogramming and differentiation of pluripotent stem cells. Reproductive Medicine and Biology, 20(1), 20–26. 33488280 10.1002/rmb2.12346 PMC7812493

[jex2115-bib-0128] Kim, D.‐K. , Nishida, H. , An, S. Y. , Shetty, A. K. , Bartosh, T. J. , & Prockop, D. J. (2016). Chromatographically isolated CD63+CD81+ extracellular vesicles from mesenchymal stromal cells rescue cognitive impairments after TBI. PNAS, 113(1), 170–175. 26699510 10.1073/pnas.1522297113 PMC4711859

[jex2115-bib-0129] Kim, B. , Li, J. , Jang, C. , & Arany, Z. (2017). Glutamine fuels proliferation but not migration of endothelial cells. Embo Journal, 36(16), 2321–2333. 28659379 10.15252/embj.201796436 PMC5556269

[jex2115-bib-0130] Kim, M. H. , Chung, C. , Oh, M. H. , Jun, J. H. , Ko, Y. , & Lee, J. H. (2021). Extracellular vesicles from a three‐dimensional culture of perivascular cells accelerate skin wound healing in a rat. Aesthetic Plastic Surgery, 45, 2437–2446. 33821312 10.1007/s00266-021-02254-y

[jex2115-bib-0131] Kim, J. Y. , Rhim, W.‐K. , Yoo, Y.‐I. , Kim, D.‐S. , Ko, K.‐W. , Heo, Y. , Park, C. G. , & Han, D. K. (2021). Defined MSC exosome with high yield and purity to improve regenerative activity. Journal of Tissue Engineering, 12, 204173142110086. 10.1177/20417314211008626 PMC8060739 33959246

[jex2115-bib-0132] King, H. W. , Michael, M. Z. , & Gleadle, J. M. (2012). Hypoxic enhancement of exosome release by breast cancer cells. BMC Cancer, 12, 421. 22998595 10.1186/1471-2407-12-421 PMC3488584

[jex2115-bib-0133] Komaki, M. , Numata, Y. , Morioka, C. , Honda, I. , Tooi, M. , Yokoyama, N. , Ayame, H. , Iwasaki, K. , Taki, A. , Oshima, N. , & Morita, I. (2017). Exosomes of human placenta‐derived mesenchymal stem cells stimulate angiogenesis. Stem Cell Research & Therapy, 8(1), 219. 28974256 10.1186/s13287-017-0660-9 PMC5627451

[jex2115-bib-0134] Kordelas, L. , Rebmann, V. , Ludwig, A.‐K. , Radtke, S. , Ruesing, J. , Doeppner, T. R. , Epple, M. , Horn, A. , Beelen, D. W. , & Giebel, B. (2014). MSC‐derived exosomes: A novel tool to treat therapy‐refractory graft‐versus‐host disease. Leukemia, 28(4), 970–973. 24445866 10.1038/leu.2014.41

[jex2115-bib-0135] Kornilov, R. , Puhka, M. , Mannerström, B. , Hiidenmaa, H. , Peltoniemi, H. , Siljander, P. , Seppänen‐Kaijansinkko, R. , & Kaur, S. (2018). Efficient ultrafiltration‐based protocol to deplete extracellular vesicles from fetal bovine serum. Journal of Extracellular Vesicles, 7(1), 1422674. 29410778 10.1080/20013078.2017.1422674 PMC5795649

[jex2115-bib-0136] Kucharzewska, P. , Christianson, H. C. , Welch, J. E. , Svensson, K. J. , Fredlund, E. , Ringnér, M. , Mörgelin, M. , Bourseau‐Guilmain, E. , Bengzon, J. , & Belting, M. (2013). Exosomes reflect the hypoxic status of glioma cells and mediate hypoxia‐dependent activation of vascular cells during tumor development. PNAS, 110(18), 7312–7317. 23589885 10.1073/pnas.1220998110 PMC3645587

[jex2115-bib-0137] Kumar, A. , & Deep, G. (2020). Hypoxia in tumor microenvironment regulates exosome biogenesis: Molecular mechanisms and translational opportunities. Cancer Letters, 479, 23–30. 32201202 10.1016/j.canlet.2020.03.017

[jex2115-bib-0138] Kwon, H. , Yang, S. , Lee, J. , Park, B. , Park, K. , Jung, J. , Bae, Y. , & Park, G. (2020). Combination treatment with human adipose tissue stem cell‐derived exosomes and fractional CO_2_ laser for acne scars: A 12‐week prospective, double‐blind, randomized, split‐face study. Acta Dermato‐Venereologica, 100(18), adv00310. 33073298 10.2340/00015555-3666 PMC9309822

[jex2115-bib-0139] Lázaro‐Ibáñez, E. , Sanz‐Garcia, A. , Visakorpi, T. , Escobedo‐Lucea, C. , Siljander, P. , Ayuso‐Sacido, Á. , & Yliperttula, M. (2014). Different gDNA content in the subpopulations of prostate cancer extracellular vesicles: Apoptotic bodies, microvesicles, and exosomes. The Prostate, 74(14), 1379–1390. 25111183 10.1002/pros.22853 PMC4312964

[jex2115-bib-0140] Liao, Z. , Jaular, L. M. , Soueidi, E. , Jouve, M. , Muth, D. C. , Schøyen, T. H. , Seale, T. , Haughey, N. J. , Ostrowski, M. , Théry, C. , & Witwer, K. W. (2019). Acetylcholinesterase is not a generic marker of extracellular vesicles. Journal of Extracellular Vesicles, 8(1), 1628592. 31303981 10.1080/20013078.2019.1628592 PMC6609367

[jex2115-bib-0141] Lin, X. , Li, S. , Wang, Y.‐J. , Wang, Y. I. , Zhong, J.‐Y. , He, J.‐Y. , Cui, X.‐J. , Zhan, J.‐K. , & Liu, Y.‐S. (2019). Exosomal Notch3 from high glucose‐stimulated endothelial cells regulates vascular smooth muscle cells calcification/aging. Life Sciences, 232, 116582. 31220525 10.1016/j.lfs.2019.116582

[jex2115-bib-0142] Lehrich, B. M. , Liang, Y. , & Fiandaca, M. S. (2021). Foetal bovine serum influence on in vitro extracellular vesicle analyses. Journal of Extracellular Vesicles, 10(3), e12061. 33532042 10.1002/jev2.12061 PMC7830136

[jex2115-bib-0143] Lei, Q. , Liu, T. , Gao, F. , Xie, H. , Sun, L. , Zhao, A. , Ren, W. , Guo, H. , Zhang, L. , Wang, H. , Chen, Z. , Guo, A.‐Y. , & Li, Q. (2017). Microvesicles as potential biomarkers for the identification of senescence in human mesenchymal stem cells. Theranostics, 7(10), 2673–2689. 28819455 10.7150/thno.18915 PMC5558561

[jex2115-bib-0144] Lehmann, B. D. , Paine, M. S. , Brooks, A. M. , Mccubrey, J. A. , Renegar, R. H. , Wang, R. , & Terrian, D. M. (2008). Senescence‐associated exosome release from human prostate cancer cells. Cancer Research, 68(19), 7864–7871. 18829542 10.1158/0008-5472.CAN-07-6538 PMC3845029

[jex2115-bib-0145] Lehrich, B. , Liang, Y. , Khosravi, P. , Federoff, H. , & Fiandaca, M. (2018). Fetal bovine serum‐derived extracellular vesicles persist within vesicle‐depleted culture media. International Journal of Molecular Sciences, 19(11), 3538. 30423996 10.3390/ijms19113538 PMC6275013

[jex2115-bib-0146] Letsiou, E. , Sammani, S. , Zhang, W. , Zhou, T. , Quijada, H. , Moreno‐Vinasco, L. , Dudek, S. M. , & Garcia, J. G. N. (2015). Pathologic mechanical stress and endotoxin exposure increases lung endothelial microparticle shedding. American Journal of Respiratory Cell and MolecularBiology, 52(2), 193–204. 10.1165/rcmb.2013-0347OC PMC4370243 25029266

[jex2115-bib-0147] Li, J. , Lee, Y. , Johansson, H. J. , Mäger, I. , Vader, P. , Nordin, J. Z. , Wiklander, O. P. B. , Lehtiö, J. , Wood, M. J. A. , & Andaloussi, S. E. (2015). Serum‐free culture alters the quantity and protein composition of neuroblastoma‐derived extracellular vesicles. Journal of Extracellular Vesicles, 4, 26883. 26022510 10.3402/jev.v4.26883 PMC4447833

[jex2115-bib-0148] Li, J. , Yawno, T. , Sutherland, A. , Loose, J. , Nitsos, I. , Allison, B. J. , Bischof, R. , Mcdonald, C. A. , Jenkin, G. , & Miller, S. L. (2017). Term vs. preterm cord blood cells for the prevention of preterm brain injury. Pediatric Research, 82(6), 1030–1038. 28723885 10.1038/pr.2017.170

[jex2115-bib-0149] Li, M. , Liao, L. , & Tian, W. (2020). Extracellular vesicles derived from apoptotic cells: An essential link between death and regeneration. Frontiers in Cell and Developmental Biology, 8, 573511. 33134295 10.3389/fcell.2020.573511 PMC7561711

[jex2115-bib-0150] Liu, J. , Qiu, X. , Lv, Y. , Zheng, C. , Dong, Y. , Dou, G. , Zhu, B. , Liu, A. , Wang, W. , Zhou, J. , Liu, S. , Liu, S. , Gao, B. , & Jin, Y. (2020). Apoptotic bodies derived from mesenchymal stem cells promote cutaneous wound healing via regulating the functions of macrophages. Stem Cell Research & Therapy, 11(1), 507. 33246491 10.1186/s13287-020-02014-w PMC7694913

[jex2115-bib-0151] Liao, Z. , Muth, D. C. , Eitan, E. , Travers, M. , Learman, L. N. , Lehrmann, E. , & Witwer, K. W. (2017). Serum extracellular vesicle depletion processes affect release and infectivity of HIV‐1 in culture. Scientific Reports, 7(1), 2558. 28566772 10.1038/s41598-017-02908-5 PMC5451420

[jex2115-bib-0152] Lötvall, J. , Hill, A. F. , Hochberg, F. , Buzás, E. I. , Di Vizio, D. , Gardiner, C. , Gho, Y. S. , Kurochkin, I. V. , Mathivanan, S. , Quesenberry, P. , Sahoo, S. , Tahara, H. , Wauben, M. H. , Witwer, K. W. , & Théry, C. (2014). Minimal experimental requirements for definition of extracellular vesicles and their functions: A position statement from the International Society for Extracellular Vesicles. Journal of Extracellular Vesicles, 3, 26913. 25536934 10.3402/jev.v3.26913 PMC4275645

[jex2115-bib-0153] Logozzi, M. , Mizzoni, D. , Angelini, D. , Di Raimo, R. , Falchi, M. , Battistini, L. , & Fais, S. (2018). Microenvironmental pH and exosome levels interplay in human cancer cell lines of different histotypes. Cancers (Basel), 10(10), 370. 30301144 10.3390/cancers10100370 PMC6210604

[jex2115-bib-0154] Lopatina, T. , Bruno, S. , Tetta, C. , Kalinina, N. , Porta, M. , & Camussi, G. (2014). Platelet‐derived growth factor regulates the secretion of extracellular vesicles by adipose mesenchymal stem cells and enhances their angiogenic potential. Cell Communication and Signaling, 12, 26. 24725987 10.1186/1478-811X-12-26 PMC4022079

[jex2115-bib-0155] Llobet, L. , Montoya, J. , López‐Gallardo, E. , & Ruiz‐Pesini, E. (2015). Side effects of culture media antibiotics on cell differentiation. Tissue Engineering Part C Methods, 21(11), 1143–1147. 26037505 10.1089/ten.TEC.2015.0062

[jex2115-bib-0156] Ludwig, N. , Razzo, B. M. , Yerneni, S. S. , & Whiteside, T. L. (2019). Optimization of cell culture conditions for exosome isolation using mini‐size exclusion chromatography (mini‐SEC). Experimental Cell Research, 378(2), 149–157. 30857972 10.1016/j.yexcr.2019.03.014

[jex2115-bib-0157] Maeshige, N. , Langston, K. , Yuan, Z.‐M. , Kondo, H. , & Fujino, H. (2021). High‐intensity ultrasound irradiation promotes the release of extracellular vesicles from C2C12 myotubes. Ultrasonics, 110, 106243. 32961400 10.1016/j.ultras.2020.106243

[jex2115-bib-0158] Masters, J. R. , Thomson, J. A. , Daly‐Burns, B. , Reid, Y. A. , Dirks, W. G. , Packer, P. , Toji, L. H. , Ohno, T. , Tanabe, H. , Arlett, C. F. , Kelland, L. R. , Harrison, M. , Virmani, A. , Ward, T. H. , Ayres, K. L. , & Debenham, G. (2001). Short tandem repeat profiling provides an international reference standard for human cell lines. Proceedings of the National Academy of Sciences, 98(14), 8012–8017. 10.1073/pnas.121616198 PMC35459 11416159

[jex2115-bib-0159] Massa, A. , Perut, F. , Chano, T. , Woloszyk, A. , Mitsiadis, T. , Avnet, S. , & Baldini, N. (2017). The effect of extracellular acidosis on the behaviour of mesenchymal stem cells in vitro. European Cells & Materials [Electronic Resource], 33, 252–267. 28368079 10.22203/eCM.v033a19

[jex2115-bib-0160] Mahmood, A. , Otruba, Z. , Weisgerber, A. W. , Palay, M. D. , Nguyen, M. T. , Bills, B. L. , & Knowles, M. K. (2023). Exosome secretion kinetics are controlled by temperature. Biophysical Journal, 122(7), 1301–1314. 36814381 10.1016/j.bpj.2023.02.025 PMC10111348

[jex2115-bib-0161] Mannerstrom, B. , Paananen, R. O. , Abu‐Shahba, A. G. , Moilanen, J. , Seppänen‐Kaijansinkko, R. , & Kaur, S. (2019). Extracellular small non‐coding RNA contaminants in fetal bovine serum and serum‐free media. Scientific Reports, 9(1), 5538. 30940830 10.1038/s41598-019-41772-3 PMC6445286

[jex2115-bib-0162] Martens‐Uzunova, E. S. , Kusuma, G. D. , Crucitta, S. , Lim, H. K. , Cooper, C. , Riches, J. E. , Azad, A. , Ochiya, T. , Boyle, G. M. , Southey, M. C. , Del Re, M. , Lim, R. , Ramm, G. A. , Jenster, G. W. , & Soekmadji, C. (2021). Androgens alter the heterogeneity of small extracellular vesicles and the small RNA cargo in prostate cancer. Journal of Extracellular Vesicles, 10(10), e12136. 34434533 10.1002/jev2.12136 PMC8374107

[jex2115-bib-0163] Mathew, S. A. , & Bhonde, R. (2017). Mesenchymal stromal cells isolated from gestationally diabetic human placenta exhibit insulin resistance, decreased clonogenicity and angiogenesis. Placenta, 59, 1–8. 29108631 10.1016/j.placenta.2017.09.002

[jex2115-bib-0164] Mckeown, S. R. (2014). Defining normoxia, physoxia and hypoxia in tumours‐implications for treatment response. The British Journal of Radiology, 87(1035), 20130676. 24588669 10.1259/bjr.20130676 PMC4064601

[jex2115-bib-0165] Mehryab, F. , Rabbani, S. , Shahhosseini, S. , Shekari, F. , Fatahi, Y. , Baharvand, H. , & Haeri, A. (2020). Exosomes as a next‐generation drug delivery system: An update on drug loading approaches, characterization, and clinical application challenges. Acta Biomaterialia, 113, 42–62. 32622055 10.1016/j.actbio.2020.06.036

[jex2115-bib-0166] Melki, I. , Tessandier, N. , Zufferey, A. , & Boilard, E. (2017). Platelet microvesicles in health and disease. Platelets, 28(3), 214–221. 28102737 10.1080/09537104.2016.1265924

[jex2115-bib-0167] Menard, C. , Pacelli, L. , Bassi, G. , Dulong, J. , Bifari, F. , Bezier, I. , Zanoncello, J. , Ricciardi, M. , Latour, M. , Bourin, P. , Schrezenmeier, H. , Sensebé, L. , Tarte, K. , & Krampera, M. (2013). Clinical‐grade mesenchymal stromal cells produced under various good manufacturing practice processes differ in their immunomodulatory properties: Standardization of immune quality controls. Stem Cells and Development, 22(12), 1789–1801. 23339531 10.1089/scd.2012.0594 PMC3668498

[jex2115-bib-0168] Mehta, I. S. , Amira, M. , Harvey, A. J. , & Bridger, J. M. (2010). Rapid chromosome territory relocation by nuclear motor activity in response to serum removal in primary human fibroblasts. Genome Biology, 11(1), R5. 20070886 10.1186/gb-2010-11-1-r5 PMC2847717

[jex2115-bib-0169] Mendt, M. , Kamerkar, S. , Sugimoto, H. , Mcandrews, K. M. , Wu, C.‐C. , Gagea, M. , Yang, S. , Blanko, E. V. R. , Peng, Q. , Ma, X. , Marszalek, J. R. , Maitra, A. , Yee, C. , Rezvani, K. , Shpall, E. , Lebleu, V. S. , & Kalluri, R. (2018). Generation and testing of clinical‐grade exosomes for pancreatic cancer. JCI Insight, 3(8), e99263. 29669940 10.1172/jci.insight.99263 PMC5931131

[jex2115-bib-0170] Merten, O.‐W. (2002). Virus contaminations of cell cultures–a biotechnological view. Cytotechnology, 39(2), 91–116. 19003296 10.1023/A:1022969101804 PMC3463984

[jex2115-bib-0171] Merckx, G. , Hosseinkhani, B. , Kuypers, S. , Deville, S. , Irobi, J. , Nelissen, I. , Michiels, L. , Lambrichts, I. , & Bronckaers, A. (2020). Angiogenic effects of human dental pulp and bone marrow‐derived mesenchymal stromal cells and their extracellular vesicles. Cells, 9(2), 312. 32012900 10.3390/cells9020312 PMC7072370

[jex2115-bib-0172] Meza‐Zepeda, L. A. , Noer, A. , Dahl, J. A. , Micci, F. , Myklebost, O. , & Collas, P. (2008). High‐resolution analysis of genetic stability of human adipose tissue stem cells cultured to senescence. Journal of Cellular and Molecular Medicine, 12(2), 553–563. 18419597 10.1111/j.1582-4934.2007.00146.x PMC3822542

[jex2115-bib-0173] Mitchell, J. P. , Court, J. , Mason, M. D. , Tabi, Z. , & Clayton, A. (2008). Increased exosome production from tumour cell cultures using the Integra CELLine Culture System. Journal of Immunological Methods, 335(1–2), 98–105. 18423480 10.1016/j.jim.2008.03.001

[jex2115-bib-0174] Miceli, V. , Pampalone, M. , Vella, S. , Carreca, A. P. , Amico, G. , & Conaldi, G. (2019). Comparison of immunosuppressive and angiogenic properties of human amnion‐derived mesenchymal stem cells between 2D and 3D culture systems. Stem Cells International, 2019, 7486279. 30911299 10.1155/2019/7486279 PMC6397962

[jex2115-bib-0175] Morrell, A. E. , Brown, G. N. , Robinson, S. T. , Sattler, R. L. , Baik, A. D. , Zhen, G. , Cao, X. , Bonewald, L. F. , Jin, W. , Kam, L. C. , & Guo, X. E. (2018). Mechanically induced Ca(2+) oscillations in osteocytes release extracellular vesicles and enhance bone formation. Bone Research, 6, 6. 29581909 10.1038/s41413-018-0007-x PMC5859015

[jex2115-bib-0176] Morse, M. A. , Garst, J. , Osada, T. , Khan, S. , Hobeika, A. , Clay, T. M. , Valente, N. , Shreeniwas, R. , Sutton, M. , Delcayre, A. , Hsu, D.‐H. , Le Pecq, J.‐B. , & Lyerly, H. K. (2005). A phase I study of dexosome immunotherapy in patients with advanced non‐small cell lung cancer. Journal of Translational Medicine, 3(1), 9. 15723705 10.1186/1479-5876-3-9 PMC551593

[jex2115-bib-0177] Moruno, F. , Pérez‐Jiménez, E. , & Knecht, E. (2012). Regulation of autophagy by glucose in Mammalian cells. Cells, 1(3), 372–395. 24710481 10.3390/cells1030372 PMC3901114

[jex2115-bib-0178] Nakamura, Y. , Miyaki, S. , Ishitobi, H. , Matsuyama, S. , Nakasa, T. , Kamei, N. , Akimoto, T. , Higashi, Y. , & Ochi, M. (2015). Mesenchymal‐stem‐cell‐derived exosomes accelerate skeletal muscle regeneration. Febs Letters, 589(11), 1257–1265. 25862500 10.1016/j.febslet.2015.03.031

[jex2115-bib-0179] Nakase, I. , Ueno, N. , Matsuzawa, M. , Noguchi, K. , Hirano, M. , Omura, M. , Takenaka, T. , Sugiyama, A. , Bailey Kobayashi, N. , Hashimoto, T. , Takatani‐Nakase, T. , Yuba, E. , Fujii, I. , Futaki, S. , & Yoshida, T. (2021). Environmental pH stress influences cellular secretion and uptake of extracellular vesicles. FEBS Open Bio, 11(3), 753–767. 10.1002/2211-5463.13107 PMC7931216 33533170

[jex2115-bib-0180] Nassar, W. , El‐Ansary, M. , Sabry, D. , Mostafa, M. A. , Fayad, T. , Kotb, E. , Temraz, M. , Saad, A.‐N. , Essa, W. , & Adel, H. (2016). Umbilical cord mesenchymal stem cells derived extracellular vesicles can safely ameliorate the progression of chronic kidney diseases. Biomaterials Research, 20, 21–21. 27499886 10.1186/s40824-016-0068-0 PMC4974791

[jex2115-bib-0181] Németh, A. , Orgovan, N. , Sódar, B. W. , Osteikoetxea, X. , Pálóczi, K. , Szabó‐Taylor, K. É. , Vukman, K. V. , Kittel, Á. , Turiák, L. , Wiener, Z. , Tóth, S. , Drahos, L. , Vékey, K. , Horvath, R. , & Buzás, E. I. (2017). Antibiotic‐induced release of small extracellular vesicles (exosomes) with surface‐associated DNA. Scientific Reports, 7(1), 8202. 28811610 10.1038/s41598-017-08392-1 PMC5557920

[jex2115-bib-0182] Nieuwland, R. , Falcón‐Pérez, J. M. , Théry, C. , & Witwer, K. W. (2020). Rigor and standardization of extracellular vesicle research: Paving the road towards robustness. Journal of Extracellular Vesicles, 10(2), e12037. 33343835 10.1002/jev2.12037 PMC7735957

[jex2115-bib-0183] Obrochta, K. M. , Krois, C. R. , Campos, B. , & Napoli, J. L. (2015). Insulin regulates retinol dehydrogenase expression and all‐trans‐retinoic acid biosynthesis through FoxO1. Journal of Biological Chemistry, 290(11), 7259–7268. 25627686 10.1074/jbc.M114.609313 PMC4358144

[jex2115-bib-0184] Ochieng, J. , Pratap, S. , Khatua, A. K. , & Sakwe, A. M. (2009). Anchorage‐independent growth of breast carcinoma cells is mediated by serum exosomes. Experimental Cell Research, 315(11), 1875–1888. 19327352 10.1016/j.yexcr.2009.03.010 PMC2742412

[jex2115-bib-0185] Oeller, M. , Laner‐Plamberger, S. , Krisch, L. , Rohde, E. , Strunk, D. , & Schallmoser, K. (2021). Human platelet lysate for good manufacturing practice‐compliant cell production. International Journal of Molecular Sciences, 22(10), 5178. 34068404 10.3390/ijms22105178 PMC8153614

[jex2115-bib-0186] Organization, W. H. (2004). Guidelines on viral inactivation and removal procedures intended to assure the viral safety of human blood plasma products. WHO Technical Report, Series, 924, 150–224.

[jex2115-bib-0187] Otsuka, K. , Yamamoto, Y. , & Ochiya, T. (2020). Uncovering temperature‐dependent extracellular vesicle secretion in breast cancer. Journal of Extracellular Vesicles, 10(2), e12049. 33408817 10.1002/jev2.12049 PMC7775566

[jex2115-bib-0188] Pachler, K. , Lener, T. , Streif, D. , Dunai, Z. A. , Desgeorges, A. , Feichtner, M. , Öller, M. , Schallmoser, K. , Rohde, E. , & Gimona, M. (2017). A Good Manufacturing Practice‐grade standard protocol for exclusively human mesenchymal stromal cell‐derived extracellular vesicles. Cytotherapy, 19(4), 458–472. 28188071 10.1016/j.jcyt.2017.01.001

[jex2115-bib-0189] Palviainen, M. , Saari, H. , Kärkkäinen, O. , Pekkinen, J. , Auriola, S. , Yliperttula, M. , Puhka, M. , Hanhineva, K. , & Siljander, R.‐M. (2019). Metabolic signature of extracellular vesicles depends on the cell culture conditions. Journal of Extracellular Vesicles, 8(1), 1596669. 31007875 10.1080/20013078.2019.1596669 PMC6461113

[jex2115-bib-0190] Palviainen, M. , Saraswat, M. , Varga, Z. , Kitka, D. , Neuvonen, M. , Puhka, M. , Joenväärä, S. , Renkonen, R. , Nieuwland, R. , Takatalo, M. , & Siljander, R. M. (2020). Extracellular vesicles from human plasma and serum are carriers of extravesicular cargo—Implications for biomarker discovery. PLoS ONE, 15(8), e0236439. 32813744 10.1371/journal.pone.0236439 PMC7446890

[jex2115-bib-0191] Pamies, D. , Bal‐Price, A. , Simeonov, A. , Tagle, D. , Allen, D. , Gerhold, D. , Yin, D. , Pistollato, F. , Inutsuka, T. , Sullivan, K. , Stacey, G. , Salem, H. , Leist, M. , Daneshian, M. , Vemuri, M. C. , McFarland, R. , Coecke, S. , Fitzpatrick, S. C. , Lakshmipathy, U. , … Hartung, T. (2017). Good cell culture practice for stem cells and stem‐cell‐derived models. Altex, 34(1), 95–132. 27554434 10.14573/altex.1607121

[jex2115-bib-0192] Pamies, D. (2018). Advanced good cell culture practice for human primary, stem cell‐derived and organoid models as well as microphysiological systems. Altex, 35(3), 353–378. 29697851 10.14573/altex.1710081

[jex2115-bib-0193] Park, S. J. , Kim, J. M. , Kim, J. , Hur, J. , Park, S. , Kim, K. , Shin, H.‐J. , & Chwae, Y.‐J. (2018). Molecular mechanisms of biogenesis of apoptotic exosome‐like vesicles and their roles as damage‐associated molecular patterns. Proceedings of the National Academy of Sciences, 115(50), E11721–E11730. 10.1073/pnas.1811432115 PMC6294905 30463946

[jex2115-bib-0194] Park, D. J. , Yun, W. S. , Kim, W. C. , Park, J.‐E. , Lee, S. H. , Ha, S. , Choi, J. S. , Key, J. , & Seo, Y. J. (2020). Improvement of stem cell‐derived exosome release efficiency by surface‐modified nanoparticles. Journal of Nanobiotechnology, 18(1), 178. 33287848 10.1186/s12951-020-00739-7 PMC7720507

[jex2115-bib-0195] Park, K. Y. , Han, H. S. , Park, J. W. , Kwon, H. H. , Park, G. H. , & Seo, S. J. (2021). Exosomes derived from human adipose tissue‐derived mesenchymal stem cells for the treatment of dupilumab‐related facial redness in patients with atopic dermatitis: A report of two cases. Journal of Cosmetic Dermatology, 21(2), 844–849. 33844417 10.1111/jocd.14153

[jex2115-bib-0196] Parolini, I. , Federici, C. , Raggi, C. , Lugini, L. , Palleschi, S. , De Milito, A. , Coscia, C. , Iessi, E. , Logozzi, M. , Molinari, A. , Colone, M. , Tatti, M. , Sargiacomo, M. , & Fais, S. (2009). Microenvironmental pH is a key factor for exosome traffic in tumor cells. Journal of Biological Chemistry, 284(49), 34211–34222. 19801663 10.1074/jbc.M109.041152 PMC2797191

[jex2115-bib-0197] Patel, D. B. , Gray, K. M. , Santharam, Y. , Lamichhane, T. N. , Stroka, K. M. , & Jay, S. M. (2017). Impact of cell culture parameters on production and vascularization bioactivity of mesenchymal stem cell‐derived extracellular vesicles. Bioengineering and Translational Medicine, 2(2), 170–179. 28932818 10.1002/btm2.10065 PMC5579732

[jex2115-bib-0198] Patel, D. B. , Santoro, M. , Born, L. J. , Fisher, J. P. , & Jay, S. M. (2018). Towards rationally designed biomanufacturing of therapeutic extracellular vesicles: Impact of the bioproduction microenvironment. Biotechnology Advances, 36(8), 2051–2059. 30218694 10.1016/j.biotechadv.2018.09.001 PMC6250573

[jex2115-bib-0199] Patel, D. B. , Luthers, C. R. , Lerman, M. J. , Fisher, J. P. , & Jay, S. M. (2019). Enhanced extracellular vesicle production and ethanol‐mediated vascularization bioactivity via a 3D‐printed scaffold‐perfusion bioreactor system. Acta Biomaterialia, 95, 236–244. 30471476 10.1016/j.actbio.2018.11.024 PMC6531369

[jex2115-bib-0200] Payr, S. , Schuseil, T. , Unger, M. , Seeliger, C. , Tiefenboeck, T. , Balmayor, E. R. , & Van Griensven, M. (2020). Effect of donor age and 3D‐cultivation on osteogenic differentiation capacity of adipose‐derived mesenchymal stem cells. Scientific Reports, 10(1), 10408. 32591595 10.1038/s41598-020-67254-5 PMC7319953

[jex2115-bib-0201] Poon, I. K. H. , Parkes, M. A. F. , Jiang, L. , Atkin‐Smith, G. K. , Tixeira, R. , Gregory, C. D. , Ozkocak, D. C. , Rutter, S. F. , Caruso, S. , Santavanond, J. P. , Paone, S. , Shi, B. , Hodge, A. L. , Hulett, M. D. , Chow, J. D. Y. , Phan, T. K. , & Baxter, A. A. (2019). Moving beyond size and phosphatidylserine exposure: Evidence for a diversity of apoptotic cell‐derived extracellular vesicles in vitro. Journal of Extracellular Vesicles, 8(1), 1608786. 31069027 10.1080/20013078.2019.1608786 PMC6493268

[jex2115-bib-0202] Pecan, P. , Hambalkó, S. , Ha, V. T. , Nagy, C. T. , Pelyhe, C. , Lainšček, D. , Kenyeres, B. , Brenner, G. B. , Görbe, A. , Kittel, Á. , Barteková, M. , Ferdinandy, P. , Manček‐Keber, M. , & Giricz, Z. (2020). calcium ionophore‐induced extracellular vesicles mediate cytoprotection against simulated ischemia/reperfusion injury in cardiomyocyte‐derived cell lines by inducing heme oxygenase 1. International Journal of Molecular Sciences, 21(20), 7687. 33081396 10.3390/ijms21207687 PMC7589052

[jex2115-bib-0203] Peltzer, J. , Lund, K. , Goriot, M. E. , Grosbot, M. , Lataillade, J. J. , Mauduit, P. , & Banzet, S. (2020). Interferon‐γ and hypoxia priming have limited effect on the miRNA landscape of human mesenchymal stromal cells‐derived extracellular vesicles. Frontiers in Cell and Developmental Biology, 8(1434), 581436. 33384991 10.3389/fcell.2020.581436 PMC7769832

[jex2115-bib-0204] Perut, F. , Roncuzzi, L. , Zini, N. , Massa, A. , & Baldini, N. (2019). Extracellular nanovesicles secreted by human osteosarcoma cells promote angiogenesis. Cancers (Basel), 11(6), 779. 31195680 10.3390/cancers11060779 PMC6627280

[jex2115-bib-0205] Pham, C. V. , Midge, S. , Barua, H. , Zhang, Y. , Ngoc‐Gia Nguyen, T. , Barrero, R. A. , Duan, A. , Yin, W. , Jiang, G. , Hou, Y. , Zhou, S. , Wang, Y. , Xie, X. , Tran, H. L. , Xiang, D. , & Duan, W. (2021). Bovine extracellular vesicles contaminate human extracellular vesicles produced in cell culture conditioned medium when ‘exosome‐depleted serum’ is utilised. Archives of Biochemistry and Biophysics, 708, 108963. 34126088 10.1016/j.abb.2021.108963

[jex2115-bib-0206] Phan, J. , Kumar, P. , Hao, D. , Gao, K. , Farmer, D. , & Wang, A. (2018). Engineering mesenchymal stem cells to improve their exosome efficacy and yield for cell‐free therapy. Journal of Extracellular Vesicles, 7(1), 1522236. 30275938 10.1080/20013078.2018.1522236 PMC6161586

[jex2115-bib-0207] Poupardin, R. , Wolf, M. , & Strunk, D. (2021). Adherence to minimal experimental requirements for defining extracellular vesicles and their functions. Advanced Drug Delivery Reviews, 176, 113872. 34284058 10.1016/j.addr.2021.113872

[jex2115-bib-0208] Phan, T. K. , Ozkocak, D. C. , & Poon, I. K. H. (2020). Unleashing the therapeutic potential of apoptotic bodies. Biochemical Society Transactions, 48(5), 2079–2088. 32869835 10.1042/BST20200225 PMC7609033

[jex2115-bib-0209] Quah, B. J. C. , & O'neill, H. C. (2007). Mycoplasma contaminants present in exosome preparations induce polyclonal B cell responses. Journal of Leukoc Biology, 82(5), 1070–1082. 10.1189/jlb.0507277 17698916

[jex2115-bib-0210] Reid, Y. , Storts, D. , Riss, T. , & Minor, L. (2004). Authentication of human cell lines by STR DNA profiling analysis. In S. Markossian (Ed.), et al., Assay guidance manual. Bethesda (MD).

[jex2115-bib-0211] Revision, U.S.P.C.C.o . (2008). United States Pharmacopeia, the National Formulary. United States Pharmacopeial Convention. Incorporated.

[jex2115-bib-0212] Ren, J. , Wang, H. , Tran, K. , Civini, S. , Jin, P. , Castiello, L. , Feng, J. , Kuznetsov, S. A. , Robey, G. , Sabatino, M. , & Stroncek, D. F. (2015). Human bone marrow stromal cell confluence: Effects on cell characteristics and methods of assessment. Cytotherapy, 17(7), 897–911. 25882666 10.1016/j.jcyt.2015.03.607 PMC4461557

[jex2115-bib-0213] Rice, G. E. , Scholz‐Romero, K. , Sweeney, E. , Peiris, H. , Kobayashi, M. , Duncombe, G. , Mitchell, M. D. , & Salomon, C. (2015). The effect of glucose on the release and bioactivity of exosomes from first trimester trophoblast cells. Journal of Clinical Endocrinology and Metabolism, 100(10), E1280–E1288. 26241326 10.1210/jc.2015-2270

[jex2115-bib-0214] Ridger, V. C. , Boulanger, C. M. , Angelillo‐Scherrer, A. , Badimon, L. , Blanc‐Brude, O. , Bochaton‐Piallat, M. L. , Boilard, E. , Buzas, E. I. , Caporali, A. , Dignat‐George, F. , Evans, C. , Lacroix, R. , Lutgens, E. , Ketelhuth, D. F. J. , Nieuwland, R. , Toti, F. , Tunon, J. , Weber, C. , & Hoefer, I. E. (2017). Microvesicles in vascular homeostasis and diseases. Position Paper of the European Society of Cardiology (ESC) Working Group on Atherosclerosis and Vascular Biology. Thromb Haemost, 117(7), 1296–1316. 28569921 10.1160/TH16-12-0943

[jex2115-bib-0215] Rocha, S. , Carvalho, J. , Oliveira, P. , Voglstaetter, M. , Schvartz, D. , Thomsen, A. R. , Walter, N. , Khanduri, R. , Sanchez, J.‐C. , Keller, A. , Oliveira, C. , & Nazarenko, I. (2019). 3D cellular architecture affects microRNA and protein cargo of extracellular vesicles. Advanced Science (Weinh), 6(4), 1800948. 10.1002/advs.201800948 PMC6382357 30828519

[jex2115-bib-0216] Rohde, E. , Pachler, K. , & Gimona, M. (2019). Manufacturing and characterization of extracellular vesicles from umbilical cord‐derived mesenchymal stromal cells for clinical testing. Cytotherapy, 21(6), 581–592. 30979664 10.1016/j.jcyt.2018.12.006

[jex2115-bib-0217] Rosenberger, L. , Ezquer, M. , Lillo‐Vera, F. , Pedraza, L. , Ortúzar, M. I. , González, L. , Figueroa‐Valdés, A. I. , Cuenca, J. , Ezquer, F. , Khoury, M. , & Alcayaga‐Miranda, F. (2019). Stem cell exosomes inhibit angiogenesis and tumor growth of oral squamous cell carcinoma. Scientific Reports, 9(1), 663–663. 30679544 10.1038/s41598-018-36855-6 PMC6345809

[jex2115-bib-0218] Rubin, H. (2019). Deprivation of glutamine in cell culture reveals its potential for treating cancer. Proceedings of the National Academy of Sciences, 116(14), 6964–6968. 10.1073/pnas.1815968116 PMC6452721 30877243

[jex2115-bib-0219] Ryu, A. H. , Eckalbar, W. L. , Kreimer, A. , Yosef, N. , & Ahituv, N. (2017). Use antibiotics in cell culture with caution: Genome‐wide identification of antibiotic‐induced changes in gene expression and regulation. Scientific Reports, 7(1), 1–9. 28790348 10.1038/s41598-017-07757-w PMC5548911

[jex2115-bib-0220] Salomon, C. , Kobayashi, M. , Ashman, K. , Sobrevia, L. , Mitchell, M. D. , & Rice, G. E. (2013). Hypoxia‐induced changes in the bioactivity of cytotrophoblast‐derived exosomes. PLoS ONE, 8(11), e79636. 24244532 10.1371/journal.pone.0079636 PMC3823597

[jex2115-bib-0221] Salomon, C. , Ryan, J. , Sobrevia, L. , Kobayashi, M. , Ashman, K. , Mitchell, M. , & Rice, G. E. (2013). Exosomal signaling during hypoxia mediates microvascular endothelial cell migration and vasculogenesis. PLoS ONE, 8(7), e68451. 23861904 10.1371/journal.pone.0068451 PMC3704530

[jex2115-bib-0222] Salomon, C. , Yee, S. , Scholz‐Romero, K. , Kobayashi, M. , Vaswani, K. , Kvaskoff, D. , Illanes, S. E. , Mitchell, M. D. , & Rice, G. E. (2014). Extravillous trophoblast cells‐derived exosomes promote vascular smooth muscle cell migration. Frontiers in Pharmacology, 5, 175. 25157233 10.3389/fphar.2014.00175 PMC4128075

[jex2115-bib-0223] Sammour, I. , Somashekar, S. , Huang, J. , Batlahally, S. , Breton, M. , Valasaki, K. , Khan, A. , Wu, S. , & Young, K. C. (2016). The effect of gender on mesenchymal stem cell (MSC) efficacy in neonatal hyperoxia‐induced lung injury. PLoS ONE, 11(10), e0164269–e0164269. 27711256 10.1371/journal.pone.0164269 PMC5053475

[jex2115-bib-0224] Sarkar, P. , Redondo, J. , Kemp, K. , Ginty, M. , Wilkins, A. , Scolding, N. J. , & Rice, C. M. (2018). Reduced neuroprotective potential of the mesenchymal stromal cell secretome with ex vivo expansion, age and progressive multiple sclerosis. Cytotherapy, 20(1), 21–28. 28917625 10.1016/j.jcyt.2017.08.007 PMC5758344

[jex2115-bib-0225] Sato, S. , Rancourt, A. , Sato, Y. , & Satoh, M. S. (2016). Single‐cell lineage tracking analysis reveals that an established cell line comprises putative cancer stem cells and their heterogeneous progeny. Scientific Reports, 6(1), 23328. 27003384 10.1038/srep23328 PMC4802345

[jex2115-bib-0226] Savina, A. , Furlán, M. , Vidal, M. , & Colombo, M. I. (2003). Exosome release is regulated by a calcium‐dependent mechanism in K562 cells. Journal of Biological Chemistry, 278(22), 20083–20090. 12639953 10.1074/jbc.M301642200

[jex2115-bib-0227] Schallmoser, K. , Henschler, R. , Gabriel, C. , Koh, M. B. C. , & Burnouf, T. (2020). Production and quality requirements of human platelet lysate: A position statement from the working party on cellular therapies of the international society of blood transfusion. Trends in Biotechnology, 38(1), 13–23. 31326128 10.1016/j.tibtech.2019.06.002

[jex2115-bib-0228] Segura, E. , Nicco, C. , Lombard, B. , Véron, P. , Raposo, G. , Batteux, F. , Amigorena, S. , & Théry, C. (2005). ICAM‐1 on exosomes from mature dendritic cells is critical for efficient naive T‐cell priming. Blood, 106(1), 216–223. 15790784 10.1182/blood-2005-01-0220

[jex2115-bib-0229] Sekiya, I. , Larson, B. L. , Smith, J. R. , Pochampally, R. , Cui, J.‐G. , & Prockop, D. J. (2002). Expansion of human adult stem cells from bone marrow stroma: Conditions that maximize the yields of early progenitors and evaluate their quality. Stem Cells, 20(6), 530–541. 12456961 10.1634/stemcells.20-6-530

[jex2115-bib-0230] Sengupta, V. , Sengupta, S. , Lazo, A. , Woods, P. , Nolan, A. , & Bremer, N. (2020). Exosomes derived from bone marrow mesenchymal stem cells as treatment for severe COVID‐19. Stem Cells and Development, 29(12), 747–754. 32380908 10.1089/scd.2020.0080 PMC7310206

[jex2115-bib-0231] Seno, K. , Tanikawa, N. , Takahashi, H. , Ohkuchi, A. , Suzuki, H. , Matsubara, S. , Iwata, H. , Kuwayama, T. , & Shirasuna, K. (2018). Oxygen concentration modulates cellular senescence and autophagy in human trophoblast cells. American Journal of Reproductive Immunology, 79(6), e12826. 29446169 10.1111/aji.12826

[jex2115-bib-0232] Shlomovitz, I. , Erlich, Z. , Arad, G. , Edry‐Botzer, L. , Zargarian, S. , Cohen, H. , Manko, T. , Ofir‐Birin, Y. , Cooks, T. , Regev‐Rudzki, N. , & Gerlic, M. (2021). Proteomic analysis of necroptotic extracellular vesicles. Cell Death & Disease, 12(11), 1059–1059. 34750357 10.1038/s41419-021-04317-z PMC8575773

[jex2115-bib-0233] Shay, J. W. , & Wright, W. E. (2000). Hayflick, his limit, and cellular ageing. Nature Reviews Molecular Cell Biology, 1(1), 72–76. 10.1038/35036093 11413492

[jex2115-bib-0234] Shekari, F. , Nazari, A. , Assar Kashani, S. , Hajizadeh‐Saffar, E. , Lim, R. , & Baharvand, H. (2021). Pre‐clinical investigation of mesenchymal stromal cell‐derived extracellular vesicles: A systematic review. Cytotherapy, 23(4), 277–284. 33541780 10.1016/j.jcyt.2020.12.009

[jex2115-bib-0235] Shelke, G. V. , Lässer, C. , Gho, Y. S. , & Lötvall, J. (2014). Importance of exosome depletion protocols to eliminate functional and RNA‐containing extracellular vesicles from fetal bovine serum. Journal of Extracellular Vesicles, 3, 24783. 10.3402/jev.v3.24783 PMC4185091 25317276

[jex2115-bib-0236] Siegel, G. , Kluba, T. , Hermanutz‐Klein, U. , Bieback, K. , Northoff, H. , & Schäfer, R. (2013). Phenotype, donor age and gender affect function of human bone marrow‐derived mesenchymal stromal cells. BMC Medicine, 11, 146–146. 23758701 10.1186/1741-7015-11-146 PMC3694028

[jex2115-bib-0237] Skubis, A. , Gola, J. , Sikora, B. , Hybiak, J. , Paul‐Samojedny, M. , Mazurek, U. , & Łos, M. (2017). Impact of antibiotics on the proliferation and differentiation of human adipose‐derived mesenchymal stem cells. International Journal of Molecular Sciences, 18(12), 2522. 29186789 10.3390/ijms18122522 PMC5751125

[jex2115-bib-0238] Soekmadji, C. , Riches, J. D. , Russell, J. , Ruelcke, J. E. , Mcpherson, S. , Wang, C. , Hovens, C. M. , Corcoran, N. M. , Hill, M. M. , & Nelson, C. C. (2017). Modulation of paracrine signaling by CD9 positive small extracellular vesicles mediates cellular growth of androgen deprived prostate cancer. Oncotarget, 8(32), 52237–52255. 28881726 10.18632/oncotarget.11111 PMC5581025

[jex2115-bib-0239] Sotiropoulou, A. , Perez, S. A. , Salagianni, M. , Baxevanis, C. N. , & Papamichail, M. (2006). Characterization of the optimal culture conditions for clinical scale production of human mesenchymal stem cells. Stem Cells, 24(2), 462–471. 16109759 10.1634/stemcells.2004-0331

[jex2115-bib-0240] Staubach, S. , Bauer, F. N. , Tertel, T. , Börger, V. , Stambouli, O. , Salzig, D. , & Giebel, B. (2021). Scaled preparation of extracellular vesicles from conditioned media. Advanced Drug Delivery Reviews, 177, 113940. 34419502 10.1016/j.addr.2021.113940

[jex2115-bib-0241] Steinman, R. A. , Wentzel, A. , Lu, Y. , Stehle, C. , & Grandis, J. R. (2003). Activation of Stat3 by cell confluence reveals negative regulation of Stat3 by cdk2. Oncogene, 22(23), 3608–3615. 12789269 10.1038/sj.onc.1206523

[jex2115-bib-0242] Subbiahanadar Chelladurai, K. , Selvan Christyraj, J. D. , Rajagopalan, K. , Yesudhason, B. V. , Venkatachalam, S. , Mohan, M. , Chellathurai Vasantha, N. , & Selvan Christyraj, J. R. S. (2021). Alternative to FBS in animal cell culture—An overview and future perspective. Heliyon, 7(8), e07686–e07686. 34401573 10.1016/j.heliyon.2021.e07686 PMC8349753

[jex2115-bib-0243] Sullivan, S. , Stacey, G. N. , Akazawa, C. , Aoyama, N. , Baptista, R. , Bedford, P. , Bennaceur Griscelli, A. , Chandra, A. , Elwood, N. , Girard, M. , Kawamata, S. , Hanatani, T. , Latsis, T. , Lin, S. , Ludwig, T. E. , Malygina, T. , Mack, A. , Mountford, J. C. , Noggle, S. , … Song, J. (2018). Quality control guidelines for clinical‐grade human induced pluripotent stem cell lines. Regenerative Medicine, 13(7), 859–866. 30205750 10.2217/rme-2018-0095

[jex2115-bib-0244] Sun, L. , Zhu, W. , Zhao, P. , Zhang, J. , Lu, Y. , Zhu, Y. , Zhao, W. , Liu, Y. , Chen, Q. , & Zhang, F. (2020). Down‐regulated exosomal microRNA‐221‐3p derived from senescent mesenchymal stem cells impairs heart repair. Frontiers in Cell and Developmental Biology, 8(263), 263. 32432109 10.3389/fcell.2020.00263 PMC7214920

[jex2115-bib-0245] Sutter, A. , Rouillard, M. E. , Alshawi, S. A. , & Crocker, S. J. (2021). Extracellular matrix influences astrocytic extracellular vesicle function in wound repair. Brain Research, 1763, 147462. 33811843 10.1016/j.brainres.2021.147462

[jex2115-bib-0246] Szvicsek, Z. , Oszvald, Á. , Szabó, L. , Sándor, G. O. , Kelemen, A. , Soós, A. Á. , Pálóczi, K. , Harsányi, L. , Tölgyes, T. , Dede, K. , Bursics, A. , Buzás, E. I. , Zeöld, A. , & Wiener, Z. (2019). Extracellular vesicle release from intestinal organoids is modulated by Apc mutation and other colorectal cancer progression factors. Cellular and Molecular Life Sciences, 76(12), 2463–2476. 31028424 10.1007/s00018-019-03052-1 PMC6529386

[jex2115-bib-0247] Takahashi, A. , Okada, R. , Nagao, K. , Kawamata, Y. , Hanyu, A. , Yoshimoto, S. , Takasugi, M. , Watanabe, S. , Kanemaki, M. T. , Obuse, C. , & Hara, E. (2017). Exosomes maintain cellular homeostasis by excreting harmful DNA from cells. Nature Communications, 8, 15287. 10.1038/ncomms15287 PMC5440838 28508895

[jex2115-bib-0248] Takasugi, M. , Okada, R. , Takahashi, A. , Virya Chen, D. , Watanabe, S. , & Hara, E. (2017). Small extracellular vesicles secreted from senescent cells promote cancer cell proliferation through EphA2. Nature Communications, 8, 15729. 10.1038/ncomms15728 PMC5467215 28585531

[jex2115-bib-0249] Taylor, J. , Jaiswal, R. , & Bebawy, M. (2017). Calcium‐calpain dependent pathways regulate vesiculation in malignant breast cells. Current Cancer Drug Targets, 17(5), 486–494. 27799031 10.2174/1568009616666161026165736

[jex2115-bib-0250] Teng, X. , Chen, L. , Chen, W. , Yang, J. , Yang, Z. , & Shen, Z. (2015). Mesenchymal stem cell‐derived exosomes improve the microenvironment of infarcted myocardium contributing to angiogenesis and anti‐inflammation. Cellular Physiology and Biochemistry, 37(6), 2415–2424. 26646808 10.1159/000438594

[jex2115-bib-0251] The Japanese pharmacopoeia . (1996). 1996: Thirteenth edition. Tokyo : Society of Japanese Pharmacopoeia : Distributed by Yakuji Nippo.

[jex2115-bib-0252] Théry, C. , Witwer, K. W. , Aikawa, E. , Alcaraz, M. J. , Anderson, J. D. , Andriantsitohaina, R. , Antoniou, A. , Arab, T. , Archer, F. , Atkin‐Smith, G. K. , Ayre, D. C. , Bach, J.‐M. , Bachurski, D. , Baharvand, H. , Balaj, L. , Baldacchino, S. , Bauer, N. N. , Baxter, A. A. , Bebawy, M. , … Zuba‐Surma, E. K. (2018). Minimal information for studies of extracellular vesicles 2018 (MISEV2018): A position statement of the International Society for Extracellular Vesicles and update of the MISEV2014 guidelines. Journal of Extracellular Vesicles, 7(1), 1535750. 30637094 10.1080/20013078.2018.1535750 PMC6322352

[jex2115-bib-0253] Thippabhotla, S. , Zhong, C. , & He, M. (2019). 3D cell culture stimulates the secretion of in vivo like extracellular vesicles. Scientific Reports, 9(1), 13012. 31506601 10.1038/s41598-019-49671-3 PMC6736862

[jex2115-bib-0254] Thom, S. R. , Bhopale, V. M. , Yu, K. , Huang, W. , Kane, M. A. , & Margolis, D. J. (2017). Neutrophil microparticle production and inflammasome activation by hyperglycemia due to cytoskeletal instability. Journal of Biological Chemistry, 292(44), 18312–18324. 28972154 10.1074/jbc.M117.802629 PMC5672053

[jex2115-bib-0255] Tosar, J. P. , Cayota, A. , Eitan, E. , Halushka, M. K. , & Witwer, K. W. (2017). Ribonucleic artefacts: Are some extracellular RNA discoveries driven by cell culture medium components? Journal of Extracellular Vesicles, 6(1), 1272832. 28326168 10.1080/20013078.2016.1272832 PMC5328325

[jex2115-bib-0256] Tracy, S. A. , Ahmed, A. , Tigges, J. C. , Ericsson, M. , Pal, A. K. , Zurakowski, D. , & Fauza, D. O. (2019). A comparison of clinically relevant sources of mesenchymal stem cell‐derived exosomes: Bone marrow and amniotic fluid. Journal of Pediatric Surgery, 54(1), 86–90. 30361074 10.1016/j.jpedsurg.2018.10.020

[jex2115-bib-0257] Trajkovic, K. , Valdez, C. , Ysselstein, D. , & Krainc, D. (2019). Fluctuations in cell density alter protein markers of multiple cellular compartments, confounding experimental outcomes. PLoS ONE, 14(2), e0211727. 30716115 10.1371/journal.pone.0211727 PMC6361456

[jex2115-bib-0258] Théry, C. , Boussac, M. , Véron, P. , Ricciardi‐Castagnoli, P. , Raposo, G. , Garin, J. , & Amigorena, S. (2001). Proteomic analysis of dendritic cell‐derived exosomes: A secreted subcellular compartment distinct from apoptotic vesicles. Journal of Immunology, 166(12), 7309–7318. 10.4049/jimmunol.166.12.7309 11390481

[jex2115-bib-0259] Torreggiani, E. , Perut, F. , Roncuzzi, L. , Zini, N. , Baglìo, S. , & Baldini, N. (2014). Exosomes: Novel effectors of human platelet lysate activity. European Cells & Materials [Electronic Resource], 28, 137–151. discussion 137. 25241964 10.22203/ecm.v028a11

[jex2115-bib-0260] Urzì, O. , Bagge, R. O. , & Crescitelli, R. (2022). The dark side of foetal bovine serum in extracellular vesicle studies. Journal of Extracellular Vesicles, 11(10), 12271. 10.1002/jev2.12271 PMC9549727 36214482

[jex2115-bib-0261] Venugopal, C. , Shamir, C. , Senthilkumar, S. , Babu, J. V. , Sonu, K. , Nishtha, K. J. , Rai, K. S. , & Dhanushkodi, A. (2017). Dosage and passage dependent neuroprotective effects of exosomes derived from rat bone marrow mesenchymal stem cells: An in vitro analysis. Current Gene Therapy, 17(5), 379–390. 29366415 10.2174/1566523218666180125091952

[jex2115-bib-0262] Vion, A.‐C. , Ramkhelawon, B. , Loyer, X. , Chironi, G. , Devue, C. , Loirand, G. , Tedgui, A. , Lehoux, S. , & Boulanger, C. M. (2013). Shear stress regulates endothelial microparticle release. Circulation Research, 112(10), 1323–1333. 23536307 10.1161/CIRCRESAHA.112.300818

[jex2115-bib-0263] Viswanathan, S. , Shi, Y. , Galipeau, J. , Krampera, M. , Leblanc, K. , Martin, I. , Nolta, J. , Phinney, D. G. , & Sensebe, L. (2019). Mesenchymal stem versus stromal cells: International Society for Cell & Gene Therapy (ISCT(R)) Mesenchymal Stromal Cell committee position statement on nomenclature. Cytotherapy, 21(10), 1019–1024. 31526643 10.1016/j.jcyt.2019.08.002

[jex2115-bib-0264] Vis, M. A. M. , Ito, K. , & Hofmann, S. (2020). Impact of culture medium on cellular interactions in in vitro co‐culture systems. Frontiers in Bioengineering and Biotechnology, 8, 911. 32850750 10.3389/fbioe.2020.00911 PMC7417654

[jex2115-bib-0265] Wang, Y.‐H. , Tao, Y. C. , Wu, D. B. , Wang, M. L. , Tang, H. , & Chen, E. Q. (2021). Cell heterogeneity, rather than the cell storage solution, affects the behavior of mesenchymal stem cells in vitro and in vivo. Stem Cell Research & Therapy, 12(1), 391. 34256842 10.1186/s13287-021-02450-2 PMC8278752

[jex2115-bib-0266] Wang, B. , Shi, Y. , Chen, J. , Shao, Z. , Ni, L. , Lin, Y. , Wu, Y. , Tian, N. , Zhou, Y. , Sun, L. , Wu, A. , Hong, Z. , Wang, X. , & Zhang, X. (2021). High glucose suppresses autophagy through the AMPK pathway while it induces autophagy via oxidative stress in chondrocytes. Cell Death & Disease, 12(6), 506. 34006821 10.1038/s41419-021-03791-9 PMC8131591

[jex2115-bib-0267] Warnecke, A. , Prenzler, N. , Harre, J. , Köhl, U. , Gärtner, L. , Lenarz, T. , Laner‐Plamberger, S. , Wietzorrek, G. , Staecker, H. , Lassacher, T. , Hollerweger, J. , Gimona, M. , & Rohde, E. (2021). First‐in‐human intracochlear application of human stromal cell‐derived extracellular vesicles. Journal of Extracellular Vesicles, 10(8), e12094. 34136108 10.1002/jev2.12094 PMC8178433

[jex2115-bib-0268] Witwer, K. W. , & Théry, C. (2019). Extracellular vesicles or exosomes? On primacy, precision, and popularity influencing a choice of nomenclature. Journal of Extracellular Vesicles, 8(1), 1648167. 31489144 10.1080/20013078.2019.1648167 PMC6711079

[jex2115-bib-0269] Witwer, K. W. , Van Balkom, B. W. M. , Bruno, S. , Choo, A. , Dominici, M. , Gimona, M. , Hill, A. F. , De Kleijn, D. , Koh, M. , Lai, R. C. , Mitsialis, S. A. , Ortiz, L. A. , Rohde, E. , Asada, T. , Toh, W. S. , Weiss, D. J. , Zheng, L. , Giebel, B. , & Lim, S. K. (2019). Defining mesenchymal stromal cell (MSC)‐derived small extracellular vesicles for therapeutic applications. Journal of Extracellular Vesicles, 8(1), 1609206. 31069028 10.1080/20013078.2019.1609206 PMC6493293

[jex2115-bib-0270] Welsh, J. A. , Van Der Pol, E. , Bettin, B. A. , Carter, D. R. F. , Hendrix, A. N. , Lenassi, M. , Langlois, M.‐A. , Llorente, A. , Van De Nes, A. S. , Nieuwland, R. , Tang, V. , Wang, L. , Witwer, K. W. , & Jones, J. C. (2020). Towards defining reference materials for measuring extracellular vesicle refractive index, epitope abundance, size and concentration. Journal of Extracellular Vesicles, 9(1), 1816641–1816641. 33062218 10.1080/20013078.2020.1816641 PMC7534292

[jex2115-bib-0271] Welford, S. M. , & Giaccia, A. J. (2011). Hypoxia and senescence: The impact of oxygenation on tumor suppression. Molecular Cancer Research, 9(5), 538–544. 21385881 10.1158/1541-7786.MCR-11-0065 PMC3096743

[jex2115-bib-0272] Wolf, M. , Poupardin, R. W. , Ebner‐Peking, P. , Andrade, A. C. , Blöchl, C. , Obermayer, A. , Gomes, F. G. , Vari, B. , Maeding, N. , Eminger, E. , Binder, H.‐M. , Raninger, A. M. , Hochmann, S. , Brachtl, G. , Spittler, A. , Heuser, T. , Ofir, R. , Huber, C. G. , Aberman, Z. , … Strunk, D. (2022). A functional corona around extracellular vesicles enhances angiogenesis, skin regeneration and immunomodulation. Journal of Extracellular Vesicles, 11(4), e12207. 35398993 10.1002/jev2.12207 PMC8994701

[jex2115-bib-0273] Xia, B. , Gao, J. , Li, S. , Huang, L. , Zhu, L. , Ma, T. , Zhao, L. , Yang, Y. , Luo, K. , Xiaowei , Shi , Mei, L. , Zhang, H. , Zheng, Y. , Lu, L. , Luo, Z. , & Huang, J. (2020). Mechanical stimulation of Schwann cells promote peripheral nerve regeneration via extracellular vesicle‐mediated transfer of microRNA 23b‐3p. Theranostics, 10(20), 8974–8995. 32802175 10.7150/thno.44912 PMC7415818

[jex2115-bib-0274] Xing, H. , Tan, J. , Miao, Y. , Lv, Y. , & Zhang, Q. (2021). Crosstalk between exosomes and autophagy: A review of molecular mechanisms and therapies. Journal of Cellular and Molecular Medicine, 25(5), 2297–2308. 33506641 10.1111/jcmm.16276 PMC7933923

[jex2115-bib-0275] Xu, J. , Camfield, R. , & Gorski, S. M. (2018). The interplay between exosomes and autophagy–partners in crime. Journal of Cell Science, 131(15), jcs215210. 30076239 10.1242/jcs.215210

[jex2115-bib-0276] Xue, C. , Shen, Y. , Li, X. , Li, B. , Zhao, S. , Gu, J. , Chen, Y. , Ma, B. , Wei, J. , Han, Q. , & Zhao, R. C. (2018). Exosomes derived from hypoxia‐treated human adipose mesenchymal stem cells enhance angiogenesis through the PKA signaling pathway. Stem Cells and Development, 27(7), 456–465. 29415626 10.1089/scd.2017.0296

[jex2115-bib-0277] Yang, C. , Chalasani, G. , Ng, Y.‐H. , & Robbins, D. (2012). Exosomes released from Mycoplasma infected tumor cells activate inhibitory B cells. PLoS ONE, 7(4), e36138. 22558358 10.1371/journal.pone.0036138 PMC3338602

[jex2115-bib-0278] Yang, Q. , Nanayakkara, G. K. , Drummer, C. , Sun, Y. , Johnson, C. , Cueto, R. , Fu, H. , Shao, Y. , Wang, L. , Yang, W. Y. , Tang, P. , Liu, L.‐W. , Ge, S. , Zhou, X.‐D. , Khan, M. , Wang, H. , & Yang, X. (2017). Low‐intensity ultrasound‐induced anti‐inflammatory effects are mediated by several new mechanisms including gene induction, immunosuppressor cell promotion, and enhancement of exosome biogenesis and docking. Frontiers in Physiology, 8, 818. 29109687 10.3389/fphys.2017.00818 PMC5660123

[jex2115-bib-0279] Yerneni, S. S. , Solomon, T. , Smith, J. , & Campbell, G. (2022). Radioiodination of extravesicular surface constituents to study the biocorona, cell trafficking and storage stability of extracellular vesicles. Biochimica et Biophysica Acta General Subjects, 1866(2), 130069. 34906563 10.1016/j.bbagen.2021.130069

[jex2115-bib-0280] You, K. , Parikh, P. , Khandalavala, K. , Wicher, S. A. , Manlove, L. , Yang, B. , Roesler, A. , Roos, B. B. , Teske, J. J. , Britt, R. D. , Pabelick, C. M. , & Prakash, Y. S. (2019). Moderate hyperoxia induces senescence in developing human lung fibroblasts. American Journal of Physiology. Lung Cellular and Molecular Physiology, 317(5), L525–L536. 31411059 10.1152/ajplung.00067.2019 PMC6879905

[jex2115-bib-0281] Umezu, T. , Imanishi, S. , Azuma, K. , Kobayashi, C. , Yoshizawa, S. , Ohyashiki, K. , & Ohyashiki, J. H. (2017). Replenishing exosomes from older bone marrow stromal cells with miR‐340 inhibits myeloma‐related angiogenesis. Blood Advances, 1(13), 812–823. 29296725 10.1182/bloodadvances.2016003251 PMC5727805

[jex2115-bib-0282] Urzì, O. , Moschetti, M. , Lässer, C. , Johansson, J. , D'Arrigo, D. , Bagge, R. O. , & Crescitelli, R. (2023). Heat inactivation of foetal bovine serum causes protein contamination of extracellular vesicles. BioRxiv, 2023.03.01.530627.

[jex2115-bib-0283] Varkouhi, A. K. , Jerkic, M. , Ormesher, L. , Gagnon, S. , Goyal, S. , Rabani, R. , Masterson, C. , Spring, C. , Chen, Z. , Gu, F. X. , Dos Santos, C. C. , Curley, G. F. , & Laffey, J. G. (2019). Extracellular vesicles from interferon‐γ–primed human umbilical cord mesenchymal stromal cells reduce *Escherichia coli*–induced acute lung injury in rats. Anesthesiology, 130(5), 778–790. 30870158 10.1097/ALN.0000000000002655

[jex2115-bib-0284] Viau, S. , Eap, S. , Chabrand, L. , Lagrange, A. , & Delorme, B. (2019). Viral inactivation of human platelet lysate by gamma irradiation preserves its optimal efficiency in the expansion of human bone marrow mesenchymal stromal cells. Transfusion, 59(3), 1069–1079. 30793328 10.1111/trf.15205

[jex2115-bib-0285] Wagner, W. , Horn, P. , Castoldi, M. , Diehlmann, A. , Bork, S. , Saffrich, R. , Benes, V. , Blake, J. , Pfister, S. , Eckstein, V. , & Ho, A. D. (2008). Replicative senescence of mesenchymal stem cells: A continuous and organized process. PLoS ONE, 3(5), e2213. 18493317 10.1371/journal.pone.0002213 PMC2374903

[jex2115-bib-0286] Wang, J. , Bonacquisti, E. E. , Brown, A. D. , & Nguyen, J. (2020). Boosting the biogenesis and secretion of mesenchymal stem cell‐derived exosomes. Cells, 9(3), 660. 32182815 10.3390/cells9030660 PMC7140620

[jex2115-bib-0287] Watchrarat, K. , Korchunjit, W. , Buranasinsup, S. , Taylor, J. , Ritruechai, P. , & Wongtawan, T. (2017). MEM α promotes cell proliferation and expression of bone marrow derived equine mesenchymal stem cell gene markers but depresses differentiation gene markers. Journal of Equine Veterinary Science, 50, 8–14.

[jex2115-bib-0288] Watson, D. C. , Bayik, D. , Srivatsan, A. , Bergamaschi, C. , Valentin, A. , Niu, G. , Bear, J. , Monninger, M. , Sun, M. , Morales‐Kastresana, A. , Jones, J. C. , Felber, B. K. , Chen, X. , Gursel, I. , & Pavlakis, G. N. (2016). Efficient production and enhanced tumor delivery of engineered extracellular vesicles. Biomaterials, 105, 195–205. 27522254 10.1016/j.biomaterials.2016.07.003 PMC7156278

[jex2115-bib-0289] Witwer, K. W. , Buzás, E. I. , Bemis, L. T. , Bora, A. , Lässer, C. , Lötvall, J. , Nolte‐‘T Hoen, E. N. , Piper, M. G. , Sivaraman, S. , Skog, J. , Théry, C. , Wauben, M. H. , & Hochberg, F. (2013). Standardization of sample collection, isolation and analysis methods in extracellular vesicle research. Journal of Extracellular Vesicles, 2(1), 20360. 10.3402/jev.v2i0.20360 PMC3760646 24009894

[jex2115-bib-0290] Wu, X. , Lin, M. , Li, Y. , Zhao, X. , & Yan, F. (2009). Effects of DMEM and RPMI 1640 on the biological behavior of dog periosteum‐derived cells. Cytotechnology, 59(2), 103–111. 19496017 10.1007/s10616-009-9200-5 PMC2698442

[jex2115-bib-0291] Wu, X. , Gao, Y. , Xu, L. , Dang, W. , Yan, H. , Zou, D. , Zhu, Z. , Luo, L. , Tian, N. , Wang, X. , Tong, Y. , & Han, Z. (2017). Exosomes from high glucose‐treated glomerular endothelial cells trigger the epithelial‐mesenchymal transition and dysfunction of podocytes. Scientific Reports, 7(1), 9371. 28839221 10.1038/s41598-017-09907-6 PMC5571220

[jex2115-bib-0292] Wu, Z. , He, D. , & Li, H. (2021). Bioglass enhances the production of exosomes and improves their capability of promoting vascularization. Bioact Mater, 6(3), 823–835. 33024902 10.1016/j.bioactmat.2020.09.011 PMC7530219

[jex2115-bib-0293] Xia, L. , Zhou, X. P. , Zhu, J. H. , Xie, X. D. , Zhang, H. , Wang, X. X. , Chen, J. Z. , & Jian, S. (2007). Decrease and dysfunction of endothelial progenitor cells in umbilical cord blood with maternal pre‐eclampsia. Journal of Obstetrics and Gynaecology Research, 33(4), 465–474. 17688613 10.1111/j.1447-0756.2007.00555.x

[jex2115-bib-0294] Xie, M. , Wu, D. , Li, G. , Yang, J. , & Zhang, Y. S. (2021). Exosomes targeted towards applications in regenerative medicine. Nano Select, 2(5), 880–908.

[jex2115-bib-0295] Yan, L. , & Wu, X. (2019). Exosomes produced from 3D cultures of umbilical cord mesenchymal stem cells in a hollow‐fiber bioreactor show improved osteochondral regeneration activity. Cell Biology and Toxicology, 36(2), 165–178. 31820164 10.1007/s10565-019-09504-5 PMC7196084

[jex2115-bib-0296] Yan, L. , & Wu, X. (2020). Exosomes produced from 3D cultures of umbilical cord mesenchymal stem cells in a hollow‐fiber bioreactor show improved osteochondral regeneration activity. Cell Biology and Toxicology, 36(2), 165–178. 31820164 10.1007/s10565-019-09504-5 PMC7196084

[jex2115-bib-0297] Yan, I. K. , Shukla, N. , Borrelli, D. A. , & Patel, T. (2018). Use of a hollow fiber bioreactor to collect extracellular vesicles from cells in culture. Methods in Molecular Biology, 1740, 35–41. 29388134 10.1007/978-1-4939-7652-2_4

[jex2115-bib-0298] Yang, Y.‐H. K. , Ogando, C. R. , Wang See, C. , Chang, T.‐Y. , & Barabino, G. A. (2018). Changes in phenotype and differentiation potential of human mesenchymal stem cells aging in vitro. Stem Cell Research Therapy, 9(1), 131. 29751774 10.1186/s13287-018-0876-3 PMC5948736

[jex2115-bib-0299] Yang, L. , Zhai, Y. , Hao, Y. , Zhu, Z. , & Cheng, G. (2020). The regulatory functionality of exosomes derived from hUMSCs in 3D culture for alzheimer's disease therapy. Small, 16(3), e1906273. 31840420 10.1002/smll.201906273

[jex2115-bib-0300] Yang, L. , Zhai, Y. , Hao, Y. , Zhu, Z. , & Cheng, G. (2019). The regulatory functionality of exosomes derived from hUMSCs in 3D culture for Alzheimer's disease therapy. Small, p. e1906273. 31840420 10.1002/smll.201906273

[jex2115-bib-0301] Yang, Y. , Knight, R. , Stephens, P. , & Zhang, Y. (2020). Three‐dimensional culture of oral progenitor cells: Effects on small extracellular vesicles production and proliferative function. Journal of Oral Pathology & Medicine, 49(4), 342–349. 31788854 10.1111/jop.12981

[jex2115-bib-0302] Yang, R. , Huang, H. , Cui, S. , Zhou, Y. , Zhang, T. , & Zhou, Y. (2020). IFN‐γ promoted exosomes from mesenchymal stem cells to attenuate colitis via miR‐125a and miR‐125b. Cell Death & Disease, 11(7), 603. 32733020 10.1038/s41419-020-02788-0 PMC7393506

[jex2115-bib-0303] Zeng, Q. , Hong, S. , Wang, X. , Cheng, Y. , Sun, J. , & Xia, W. (2019). Regulation of exosomes secretion by low‐intensity pulsed ultrasound in lung cancer cells. Experimental Cell Research, 383(1), 111448. 31152706 10.1016/j.yexcr.2019.05.029

[jex2115-bib-0304] Zhang, W. , Zhou, X. , Yao, Q. , Liu, Y. , Zhang, H. , & Dong, Z. (2017). HIF‐1‐mediated production of exosomes during hypoxia is protective in renal tubular cells. American Journal of Physiology. Renal Physiology, 313(4), F906–F913. 28679592 10.1152/ajprenal.00178.2017 PMC5668579

[jex2115-bib-0305] Zhang, Q. , Fu, L. , Liang, Y. , Guo, Z. , Wang, L. , Ma, C. , & Wang, H. (2018). Exosomes originating from MSCs stimulated with TGF‐β and IFN‐γ promote Treg differentiation. Journal of Cellular Physiology, 233(9), 6832–6840. 29336475 10.1002/jcp.26436 PMC13482522

[jex2115-bib-0306] Zhang, G. , Zhu, Z. , Wang, H. , Yu, Y. , Chen, W. , Waqas, A. , Wang, Y. , & Chen, L. (2020). Exosomes derived from human neural stem cells stimulated by interferon gamma improve therapeutic ability in ischemic stroke model. Journal of Advanced Research, 24, 435–445. 32551140 10.1016/j.jare.2020.05.017 PMC7289755

[jex2115-bib-0307] Zhang, X. , Wang, N. , Huang, Y. , Li, Y. , Li, G. , Lin, Y. , Atala, A. J. , Hou, J. , & Zhao, W. (2021). Extracellular vesicles from three dimensional culture of human placental mesenchymal stem cells ameliorated renal ischemia/reperfusion injury. The International Journal of Artificial Organs, p. 391398820986809. 10.1177/0391398820986809 33467948

[jex2115-bib-0308] Zhao, Z. , Qu, L. , Shuang, T. , Wu, S. , Su, Y. , Lu, F. , Wang, D. , Chen, B. , & Hao, Q. (2020). Low‐intensity ultrasound radiation increases exosome yield for efficient drug delivery. Journal of Drug Delivery Science and Technology, 57.

[jex2115-bib-0309] Zhao, Y. , Albrecht, E. , Stange, K. , Li, Z. , Schregel, J. , Sciascia, Q. L. , Metges, C. C. , & Maak, S. (2021). Glutamine supplementation stimulates cell proliferation in skeletal muscle and cultivated myogenic cells of low birth weight piglets. Scientific Reports, 11(1), 13432. 34183762 10.1038/s41598-021-92959-6 PMC8239033

[jex2115-bib-0310] Zheng, C. , Sui, B. , Zhang, X. , Hu, J. , Chen, J. , Liu, J. , Wu, D. , Ye, Q. , Xiang, L. , Qiu, X. , Liu, S. , Deng, Z. , Zhou, J. , Liu, S. , Shi, S. , & Jin, Y. (2021). Apoptotic vesicles restore liver macrophage homeostasis to counteract type 2 diabetes. Journal of Extracellular Vesicles, 10(7), e12109. 34084287 10.1002/jev2.12109 PMC8144839

[jex2115-bib-0311] Zhou, S. , Fang, J. , Hu, M. , Pan, S. , Liu, D. , Xing, G. , & Liu, Z. (2021). Determining the influence of high glucose on exosomal lncRNAs, mRNAs, circRNAs and miRNAs derived from human renal tubular epithelial cells. Aging (Albany NY), 13(6), 8467–8480. 33714195 10.18632/aging.202656 PMC8034913

[jex2115-bib-0312] Zhu, Q.‐J. , Zhu, M. , Xu, X.‐X. , Meng, X.‐M. , & Wu, Y.‐G. (2019). Exosomes from high glucose‐treated macrophages activate glomerular mesangial cells via TGF‐beta1/Smad3 pathway in vivo and in vitro. Faseb Journal, 33(8), 9279–9290. 31162940 10.1096/fj.201802427RRR

[jex2115-bib-0313] Zhu, D. , Kusuma, G. D. , Schwab, R. , Chan, S. T. , Tan, J. , Saad, M. I. , Leeman, K. T. , Kim, C. , Wallace, E. M. , & Lim, R. (2020). Prematurity negatively affects regenerative properties of human amniotic epithelial cells in the context of lung repair. Clinical Science (London, England: 1979), 134(20), 2665–2679. 33000862 10.1042/CS20200859

[jex2115-bib-0314] Zhu, D. , Fang, H. , Kusuma, G. D. , Schwab, R. , Barabadi, M. , Chan, S. T. , Mcdonald, H. , Leong, C. M. , Wallace, E. M. , Greening, D. W. , & Lim, R. (2021). Impact of chemically defined culture media formulations on extracellular vesicle production by amniotic epithelial cells. Proteomics, 21, e2000080. 34081834 10.1002/pmic.202000080

[jex2115-bib-0315] Zou, W. , Lai, M. , Zhang, Y. , Zheng, L. , Xing, Z. , Li, T. , Zou, Z. , Song, Q. , Zhao, X. , Xia, L. , Yang, J. , Liu, A. , Zhang, H. , Cui, Z.‐K. , Jiang, Y. , & Bai, X. (2019). Exosome Release Is Regulated by mTORC1. Advanced Science (Weinh), 6(3), 1801313. 10.1002/advs.201801313 PMC6364500 30775228

